# Species-Level Deconvolution of Metagenome Assemblies with Hi-C–Based Contact Probability Maps

**DOI:** 10.1534/g3.114.011825

**Published:** 2014-05-22

**Authors:** Joshua N. Burton, Ivan Liachko, Maitreya J. Dunham, Jay Shendure

**Affiliations:** Department of Genome Sciences, University of Washington, Seattle, Washington 98195-5065

**Keywords:** Hi-C, metagenome assembly, metagenomics, clustering algorithms

## Abstract

Microbial communities consist of mixed populations of organisms, including unknown species in unknown abundances. These communities are often studied through metagenomic shotgun sequencing, but standard library construction methods remove long-range contiguity information; thus, shotgun sequencing and *de novo* assembly of a metagenome typically yield a collection of contigs that cannot readily be grouped by species. Methods for generating chromatin-level contact probability maps, *e.g.*, as generated by the Hi-C method, provide a signal of contiguity that is completely intracellular and contains both intrachromosomal and interchromosomal information. Here, we demonstrate how this signal can be exploited to reconstruct the individual genomes of microbial species present within a mixed sample. We apply this approach to two synthetic metagenome samples, successfully clustering the genome content of fungal, bacterial, and archaeal species with more than 99% agreement with published reference genomes. We also show that the Hi-C signal can secondarily be used to create scaffolded genome assemblies of individual eukaryotic species present within the microbial community, with higher levels of contiguity than some of the species’ published reference genomes.

All ecosystems on this planet include communities of microbial organisms (Howe *et al.* 2014; Xin *et al.* 2009; Hug *et al.* 2013; Venter *et al.* 2004; Renouf *et al.* 2007), including our own bodies (Qin *et al.* 2010; Huttenhower *et al.* 2012). However, our understanding of microbial communities is limited by our ability to discern which microbial taxa they contain and how these taxa contribute to community-scale phenotypes. Most microbial taxa cannot be cultured independently of their native communities (Rinke *et al.* 2013) and therefore are not readily isolated for individual analysis, *e.g.*, by genome sequencing. Such unculturable taxa may be difficult to study even if they are abundant (Iverson *et al.* 2012). Consequently, many analyses of microbial communities must treat them as a single sample, for example, by shotgun sequencing of a metagenome (Iverson *et al.* 2012, Huttenhower *et al.* 2012; Venter *et al.* 2004; Howe *et al.* 2014) or metatranscriptome (Frias-Lopez *et al.* 2008; David *et al.* 2014).

A central challenge in analyzing a metagenome involves determining which sequence reads and/or sequence contigs originated from the same taxon (Carr *et al.* 2013). Many computational methods have been developed to deconvolute metagenomic assemblies by mapping reads or contigs to assembled microbial genomes (Wood and Salzberg 2014) or by analyzing base composition (Saeed *et al.* 2012) or gene abundance (Hug *et al.* 2013; Carr *et al.* 2013). However, these strategies are handicapped by the remarkable variety of unculturable species in virtually all microbial communities and the fact that most of these species have not yet been sequenced in isolation (Howe *et al.* 2014). Individual microbial genomes have been deconvoluted from shotgun metagenome reads using methods such as mate-pair libraries (Iverson *et al.* 2012; Mitra *et al.* 2013), lineage-specific probes (Narasingarao *et al.* 2012), single-cell sequencing (Rinke *et al.* 2013), neural networks (Dick *et al.* 2009; Hug *et al.* 2013; Sharon *et al.* 2013), and differential coverage binning (Sharon *et al.* 2013; Albertsen *et al.* 2013). Some *de novo* assembly software has also been adapted to anticipate metagenomic shotgun sequence data (Peng *et al.* 2012; Namiki *et al.* 2012). These methods have succeeded in isolating whole genomes from abundant organisms in some communities, but they are specific to the communities for which they have been devised and often require prior knowledge of the community’s composition (Iverson *et al.* 2012). Metagenomic analyses would benefit greatly from a more generalizable methodology that can identify the sequence content belonging to each taxon without any *a priori* knowledge of the genomes of these organisms, especially the genomes of low-abundance taxa. Related to the challenge of determining which contigs belong to the same species are the problems of how to further define and assemble the one or multiple chromosomes that comprise each species’ genome, and how to define and assign plasmid content to one or multiple species.

To enable robust reconstruction of individual genomes from within a complex microbial community, additional information beyond standard shotgun sequencing libraries is required. We speculated that contact probability maps generated through chromosome conformation capture methods (Dekker *et al.* 2013) might inform the species-level deconvolution of metagenome assemblies. One specific method for generating contact probability maps, Hi-C, uses proximity ligation and massively parallel sequencing to generate paired-end sequence reads that capture three-dimensional genomic interactions within a cell (Lieberman-Aiden *et al.* 2009). We and others recently exploited the distance dependence of intrachromosomal interactions in Hi-C datasets to facilitate chromosome-scale *de novo* assembly of complex genomes (Burton *et al.* 2013; Kaplan and Dekker 2013). As an additional feature, because crosslinking occurs prior to cell lysis in the Hi-C protocol, each Hi-C interaction involves a pair of reads originating from within the same cell. We speculated that in the context of heterogeneous cell populations (*e.g.*, microbial communities), such pairings might inform the clustering of genome sequences originating from the same species. Importantly, the efficacy of the Hi-C protocol has recently been demonstrated in bacteria (Umbarger *et al.* 2011; Le *et al.* 2013), implying that this method could be applicable to metagenome samples containing both prokaryotic and eukaryotic cells.

Here, we provide experimental proof-of-concept for this strategy in several contexts while also describing an algorithm for this task, MetaPhase (Figure 1). We reconstruct the genomes of as many as 18 species from a single synthetic mixture of eukaryotes and/or prokaryotes, including some species with as much as 90% sequence identity to one another, and we generate high-contiguity *de novo* assemblies for individual eukaryotic species present within the synthetic microbial community. In the process, we also present the first demonstration of Hi-C in an archaeal species.

**Figure 1:**
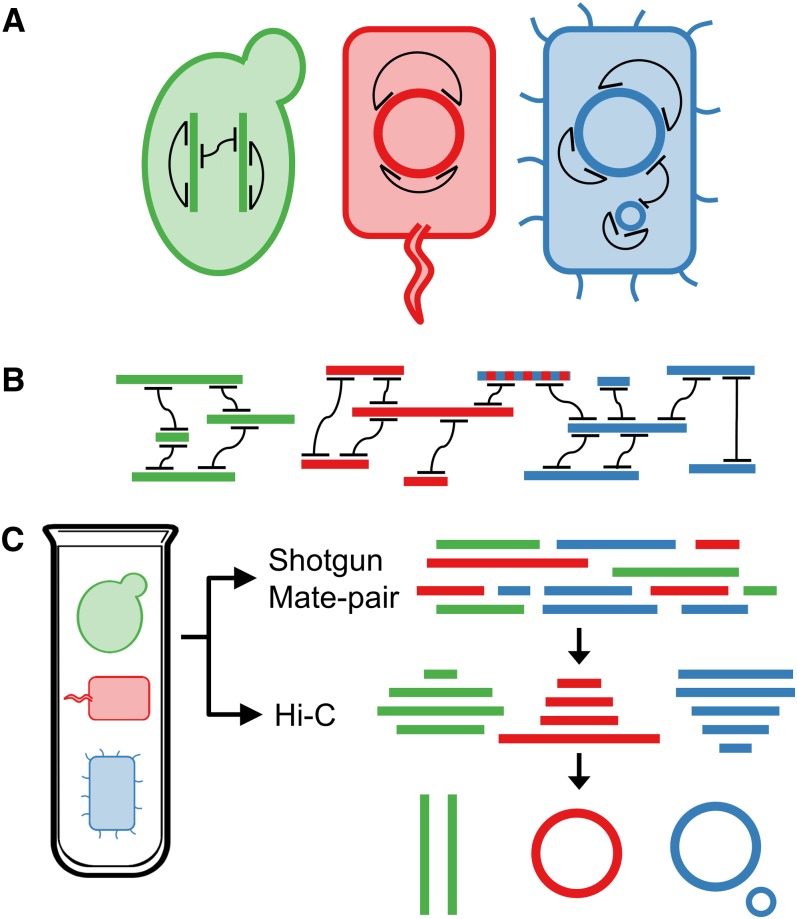
Overview of MetaPhase methodology. (A) Performing Hi-C on a mixed cell population. Shown are three microbial cells of different species (green, red, blue) with their genomes (thick colored lines or circles), which may or may not include multiple chromosomes or plasmids. A Hi-C library is prepared and sequenced from this sample. The Hi-C read pairs from this library (black lines) represent pairs of sequences that necessarily occur within the same cell. (B) Using Hi-C reads to deconvolute individual species’ genomes. A shotgun sequencing library from the same sample is used to create a draft *de novo* metagenome assembly, which contains contigs from all species (thick lines). The Hi-C reads are then aligned to this assembly. Because sequences connected by Hi-C links must appear in the same species, the contigs form clusters representing each species. Note that some sequences (*e.g.*, blue/red dotted line) may appear in multiple species, confounding the clustering. (C) MetaPhase workflow. A single metagenome sample is used to create shotgun, Hi-C, and (optionally) mate-pair libraries, which are used together to create individual species assemblies.

## Materials and Methods

### Sample collection

Cultures of individual strains listed in Table 1, Supporting Information, Table S1 (M-Y) and Table S2 (M-3D) were grown to saturation in rich media (YPD for yeasts, LB for bacteria, McCas media for *M. maripaludis*, PMsul media for *R. palustris*). Culture densities were measured by spectrophotometry and FACS. After mixing the strains, cultures were diluted with YPD media (M-Y) or with LB media (M-3D) to a final OD600 of 1.0 in a final volume of 500 mL. Formaldehyde was added to a final concentration of 1% and cultures were incubated at room temperature for 30 min. To quench the crosslinker, 5 g of glycine was added to each 500 mL of culture, and the cultures were incubated for 30 min at room temperature. Cultures were centrifuged to pellet all cells. Cell pellets were frozen at −20° until further processing.

**Table 1:** Contents of the metagenome samples analyzed

Acronym	Description	Number of Species	Species
**M-Y**	Mixture of yeasts	13	*S. cerevisiae*, other Saccharomyces; Lachancea, Kluyveromyces, etc. (Table S1)
**M-3D**	Mixture of 3 domains	18	8 yeasts (Dikarya); 9 bacteria; 1 archaeon (Table S2)

### Shotgun and mate-pair libraries

Total DNA was isolated from cultures using a standard phenol/chloroform glass bead purification followed by ethanol precipitation and subsequent cleanup using the DNA Clean and Concentrator-5 Kit (Zymo Research). Shotgun libraries were prepared using the Nextera DNA Sample Preparation Kit (Illumina). Mate-pair libraries were constructed using the Nextera Mate Pair Sample Preparation Kit (Illumina).

### Hi-C libraries

Cell pellets (∼100 μL volume each) were resuspended in 2 mL of 1× TBS buffer containing 1% Triton-X and Protease Inhibitors (cOmplete, EDTA-free; Roche) and split equally into two separate 2-mL tubes; 300–500 μL of 0.5-mm diameter glass beads were added to each tube and tubes were vortexed on the highest setting in four 5-min increments, each separated by 2-min incubations on ice. Lysate was transferred to fresh tubes. Crosslinked chromatin was recovered by centrifugation at 13 KRPM in an accuSpin Micro17 centrifuge (Fisher) and rinsed with 1× TBS buffer. Chromatin from each tube was digested overnight with 100 units of either *Hin*dIII or *Nco*I restriction endonuclease (NEB) at 37° in a total volume of 200 μL. To enrich for long-range interactions (M-3D library only), digested chromatin was centrifuged for 10 min at 13 KRPM, rinsed in 200 μL of 1× NEBuffer 2 (NEB), centrifuged again, and resuspended in 200 μL of 1× NEBuffer 2 (NEB). Restriction fragment overhangs were filled in using biotinylated dCTP (Invitrogen) and Klenow (NEB) as described (van Berkum *et al.* 2010). DNA concentration within the chromatin suspension was quantitated using the QuBit fluorometer (Invitrogen), and for each sample an 8-mL ligation reaction was set-up at a final DNA concentration of 0.5 ng/μL using T4 DNA Ligase (NEB). Ligation reactions were incubated at room temperature for 4 hr and then overnight at 70° to reverse crosslinks. DNA was purified using a standard phenol/chloroform purification followed by ethanol precipitation and resuspended in 600 μL of water with 1× NEBuffer 2 (NEB) and 1× BSA (NEB). To remove biotin from unligated DNA ends, 20 units of T4 Polymerase (NEB) were added to each 600 μL DNA sample and incubated at 25° for 10 min followed by 12° for 1 hr. DNA was purified using the DNA Clean and Concentrator-5 Kit (Zymo Research). Illumina libraries were constructed as described (van Berkum *et al.* 2010) using reagents from the Illumina Mate Pair Sample Preparation Kit. Paired-end sequencing was performed using the HiSeq and MiSeq Illumina platforms (Table 2).

**Table 2:** Sequencing libraries used in MetaPhase analyses

**Sample**	**Library Type**	**Read Length, bp**	**Read Pairs, millions**
**M-Y**	Shotgun	101	85.7
	Mate-pair	100	9.2
	Hi-C	100	81.0
**M-3D**	Hi-C	101	14.3

Hi-C libraries were all prepared with the *Hin*dIII restriction enzyme. For descriptions of sample names, see Table 1.

### Draft metagenome assembly for M-Y and M-3D

To create draft metagenome assemblies for the synthetic samples, we assembled the fragment library using the IDBA-UD assembler (Peng *et al.* 2012). We ran IDBA-UD with the –read option set to the fragment reads and the following additional parameters: ‘–pre_correction –mink 20 –maxk 60 –step 10’. We used the assembly in contig.fa rather than scaffold.fa to reduce the risk of false joins made at the scaffolding stage.

### Aligning Hi-C reads

We aligned the Hi-C reads to the draft metagenome assembly in a multi-step process. First, the reads were aligned using BWA (Li and Durbin 2009) with the option ’-n 0’, requiring a perfect match of the entire 100-bp read. For read pairs in which an alignment was not found for both reads, the reads were trimmed from 100 bp to 75 bp and were aligned using ’-n 0’ again. For read pairs in which alignment was still not found for both reads, the reads were trimmed to 50 bp and aligned using ’-n 0’ again. All read pairs for which no alignment was found were discarded from further analysis. Read pairs were also discarded if the reads did not both align within 500 bp of a restriction site, as recommended by Yaffe and Tanay (2011).

### Clustering contigs by species

To cluster the contigs of the draft metagenome assembly into individual species, we used a hybrid clustering algorithm. A graph was built, with each node representing one contig and each edge between nodes having a weight equal to the number of Hi-C read pairs linking the two contigs, normalized by the number of restriction sites on the contigs. Only the single largest component in the graph was used; the other components, generally comprising isolated contigs containing a small fraction of the total sequence length, were discarded and the contigs were not clustered. Within this component, the Jarvis-Patrick nearest-neighbor clustering algorithm (Jarvis and Patrick 1973) was applied with *k* = 100, removing some edges and reweighting all other edge weights by the frequency of their nodes’ shared nearest neighbors. This nearest-neighbor approach accounts for the likely possibility that the clusters representing each species will have different internal densities of Hi-C links due to species’ differing abundances in the sample or differing susceptibility to the cell lysis step of Hi-C. Finally, the nodes were merged together using hierarchical agglomerative clustering with an average-linkage metric (Eisen *et al.* 1998), which was applied until the number of clusters was reduced to the expected or predicted number of individual species (12 for M-Y, not including *P. pastoris*; 18 for M-3D).

### Scaffolding of genomic content within individual clusters

To scaffold the individual species’ genomes represented in each cluster of contigs, we aligned the Hi-C reads to these contigs and ran them through our Lachesis software (Burton *et al.* 2013) to create chromosome-scale scaffolds. The number of chromosomes in each species [7 for *K. wickerhamii* (Belloch *et al.* 1998); 8 for *S. stipitis* (Jeffries *et al.* 2007)] was provided as an input to Lachesis.

### Validation

To determine the true species identity of the contigs in the draft metagenome assembly, we aligned them to a combined reference genome that included the reference genomes of all strains known to be in the metagenome sample (16 strains for M-Y; 18 species for M-3D). The alignment was performed by BLASTn (Altschul *et al.* 1990) with the following stringent parameters: ’-perc_identity 95 -evalue 1e-30 -word_size 50’. A contig was defined as aligning to a species if any alignment of the contig to the species’ reference genome was found; the placement of the alignment was ignored.

## Results

### Deconvoluting yeast genomes from a synthetic mixture

**Figure 2:**
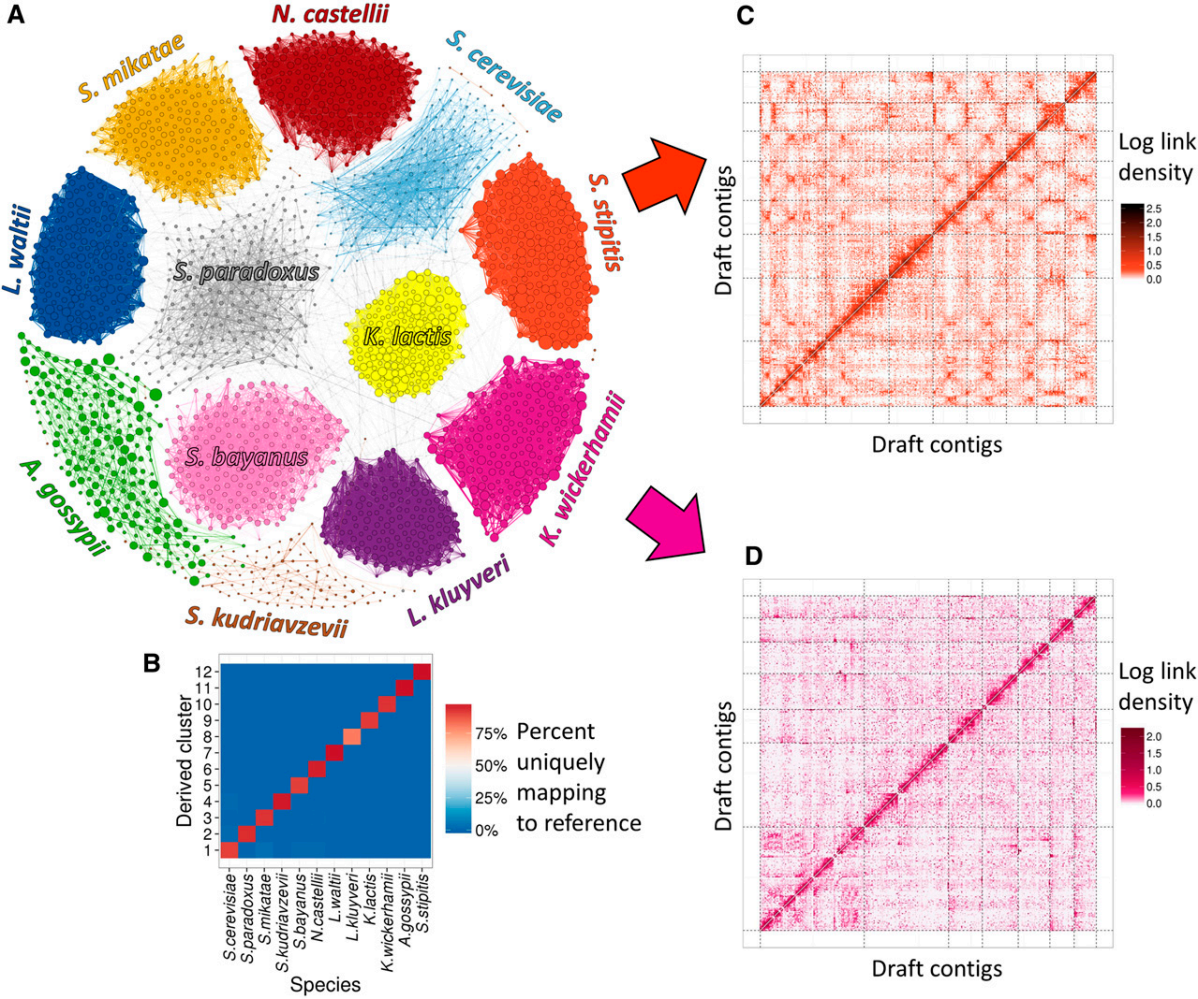
MetaPhase clustering results on the M-Y draft metagenome assembly. (A) Using Hi-C links to cluster contigs into 12 clusters, one for every species with a substantial presence in the draft assembly. Each contig is shown as a dot, with size indicating contig length, colored by species. Edge widths represent the densities of Hi-C links between the contigs shown. Only 2400 contigs are shown: the 200 largest contigs that map uniquely to each species. (B) Validation. This heatmap indicates what fraction of the sequence in each MetaPhase cluster maps uniquely to each of the reference genomes of the 12 present yeast species. Note that not all sequence is expected to map uniquely to one species. *x*-axis: the 12 yeast species. *y*-axis: the MetaPhase clusters. (C and D) Lachesis (Burton *et al.* 2013) reconstruction of individual species’ genomes within the M-Y metagenome assembly. These heatmaps show the Hi-C link density among the contigs in the MetaPhase clusters corresponding to *S. stipitis* (C) and *K. wickerhamii* (D). The *x*-axis and *y*-axis show the clustering and ordering of contigs by Lachesis. Dotted black lines demarcate chromosomal clusters. Note the expected signals of enrichment within each chromosome and on the main diagonal. The assembly in (C) is similar to the *S. stipitis* reference genome (Figure S7), whereas the assembly in (D) has far higher chromosome-scale contiguity than the best available *K. wickerhamii* reference (Baker *et al.* 2011).

To evaluate the effectiveness of the proposed strategy, we first applied it to a sample of defined, exclusively eukaryotic composition. Specifically, we created a synthetic metagenome sample consisting of 16 yeast strains (“M-Y”) (Figure 2 and Table 1) The strains include four strains of *Saccharomyces cerevisiae* as well as 12 other species of Ascomycetes at varying genetic distances from *S. cerevisiae*, all of which have publicly available reference genomes (Table S1, Figure S1, and Figure S2). These strains were grown individually to saturation in YPD medium and mixed in approximately similar proportions (with the exceptions of *S. kudriavzevii* and *P. pastoris*, which were mixed in at a much lower proportion to test the sensitivity of this approach). The mixed cell culture was treated with the cross-linking agent, formaldehyde, immediately after mixing the individual strains. Total DNA was isolated from the mixed population culture and prepared for sequencing. This resulted in 92.1 M Illumina read pairs from one shotgun library, 9.2 M Illumina read pairs from one mate-pair library, and 81.0 M read pairs from one Hi-C library (Table 2).

We used the shotgun and mate-pair (∼4 kb) read pairs to generate a draft *de novo* metagenome assembly using IDBA-UD (Peng *et al.* 2012) (see *Materials and Methods*). This assembly had 48,511 contigs with a total length of 136 Mb and an N50 contig length of 17.3 kb. Contigs from this assembly covered most of the reference genomes of all 13 yeast species present (average = 96.0%), with the exception of *P. pastoris* (13.7%), which also had a very low fraction of shotgun reads aligning to it (1.2%), confirming its low abundance in the sample (Figure S3).

We next aligned the Hi-C read pairs to the M-Y metagenome assembly, yielding a network of contigs joined by Hi-C links (Figure 2A). Then, exploiting the fact that sequences connected by Hi-C links are overwhelmingly expected to derive from the same cell, we used the links to cluster these contigs, applying a novel algorithm that combines the steps of Jarvis-Patrick clustering (Jarvis and Patrick 1973) and agglomerative hierarchical clustering (Eisen *et al.* 1998) (see *Materials and Methods*). Our algorithm suggested the presence of 12 distinct clusters in the sample based on the metric of intracluster link enrichment (Figure S4). It clustered the majority of the metagenome assembly (111 Mb or 82.2% of total sequence length) into these 12 clusters. Of the remaining 24.1 Mb of sequence not clustered, the majority (99.7%) belonged to contigs that contained no *Hin*dIII sites and thus are not expected to produce a Hi-C signal in this experiment. Bootstrapping tests confirmed the robustness of our clustering method (Table S3). The 12 clusters match closely with the 12 distinct species present in the draft assembly (excluding *P. pastoris*), and 99.2% of sequence was placed into the cluster representing a species to which it truly belongs (Figure 2B and Figure S5), allowing for the possibility of a given contig belonging to multiple species.

Further analysis of the clusters demonstrated several strengths and limitations of our method. Some species had greater Hi-C link densities than others after correcting for differences in species abundances (Figure S6). This suggests that some species’ cells are more susceptible to lysis during Hi-C than others, and MetaPhase is robust to these differences. However, distantly related species proved easier to separate than closely related species. For example, in the cluster representing *Scheffersomyces stipitis*, 99.88% of the contigs (by length) matched the *S. stipitis* reference genome; however, in the cluster representing *S. cerevisiae*, 3.3% of the contigs (by length) instead aligned uniquely to the genome of closely related *S. mikatae*. We also noted that the sequence content in the *S. cerevisiae* cluster included the contigs that aligned to any of the four *S. cerevisiae* strains’ reference genomes. This indicates that although our method is generally successful in merging closely related strains of the same species into a single cluster, genetic variation between strains causes fragmentation of the species’ sequence contigs in the metagenome assembly (Figure S3), which in turn hampers our ability to delineate this cluster correctly because smaller contigs produce a weaker and noisier Hi-C signal. Separating this cluster into sub-clusters representing each *S. cerevisiae* strain represents an additional challenge that will require further algorithmic development.

We next sought to scaffold the genomic content of individual yeast species from the clusters of contigs representing each species. We ran the contigs in each cluster through our Lachesis software (Burton *et al.* 2013) to create chromosome-scale scaffolds. With the *S. stipitis* contig cluster, this approach yielded a scaffold for each of the eight *S. stipitis* chromosomes, with a total scaffolded sequence length of 14.2 Mb (91.7% of the *S. stipitis* reference genome and 95.1% of the portion of the *S. stipitis* genome that appeared in the draft metagenome assembly) (Figure 2C). These scaffolds matched the reference *S. stipitis* genome assembly fairly well (Figure S7). There were a number of clustering errors, including one chromosomal cluster containing telomeric sequence from four other chromosomes, but the local misassembly rates were quite low: 0.9% and 1.1% for ordering and orientation errors, respectively. We applied this same method to the contig cluster representing *K. wickerhamii*, producing chromosome-scale scaffolds for each of the seven *K. wickerhamii* chromosomes, with a total length of 9.4 Mb (Figure 2D). These scaffolds, although we emphasize they have not been thoroughly validated, may represent a draft assembly with far higher contiguity than the existing *K. wickerhamii* reference genome (Baker *et al.* 2011), which has an N50 contig size of only 36.7 kb. Thus, the MetaPhase approach can be combined with Lachesis to create high-contiguity *de novo* genome assemblies of individual eukaryotic species within metagenome samples.

### Concurrently deconvoluting eukaryotic, bacterial, and archaeal genomes

We next asked whether MetaPhase could be applied to deconvolute a metagenome consisting of both eukaryotic and prokaryotic species. Toward a proof of concept, we gathered samples of 18 species including eight yeasts, nine bacteria, and one archaeon, thus representing all three domains of life (“M-3D”) (Table 1 and Figure S8). The species were grown in appropriate rich media and mixed together in similar proportions. The proportions were estimated by a combination of spectrophotometric, flow sorting, and counting approaches and were later confirmed by sequence coverage (Table S2).

**Figure 3:**
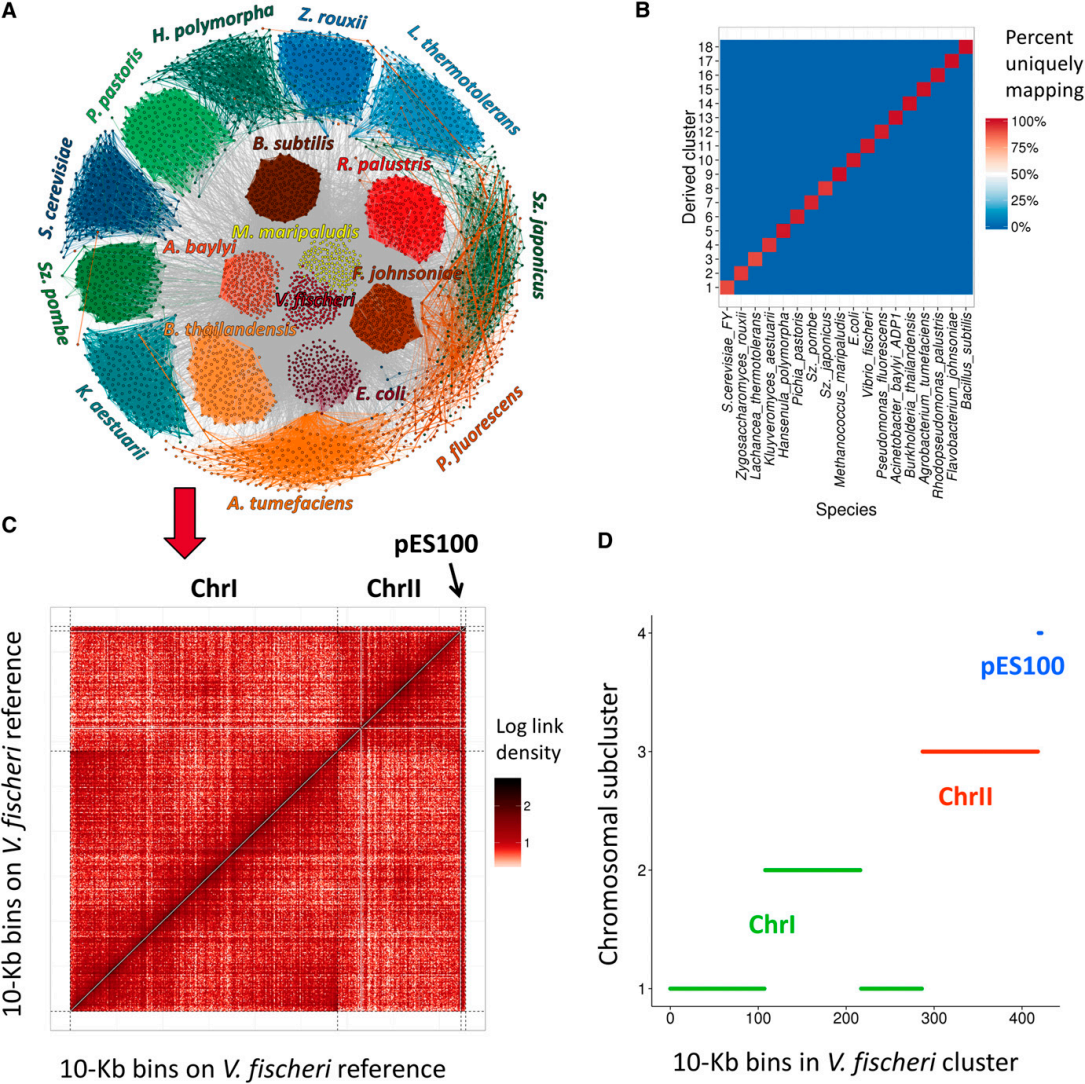
MetaPhase clustering results on the M-3D simulated contig assembly. (A) The reference genomes of the 18 species from the M-3D sample were split into 10-kb bins. Hi-C links from the metagenome sample were then used to divide the bins into 18 clusters, one for every species. The contigs are illustrated as in Figure 2A. Blue and green colors are yeast species; yellow is archaea; and red and orange are bacteria. (B) Validation. This heatmap has the same key as Figure 2B. (C) Heatmap of the M-3D Hi-C links aligned to the reference genome of *Vibrio fischeri*, one of the bacteria in the sample. The *V. fischeri* genome contains two chromosomes and a 46-kb plasmid, pES100 (demarcated by dotted black lines.) This heatmap has a resolution of 10 kb. (D) Applying Lachesis’ clustering algorithm to the *V. fischeri* clustered genome to deconvolute the pES100 plasmid from the *V. fischeri* chromosomes. The *x*-axis shows the 424 simulated contigs in the *V. fischeri* cluster derived in (A and B). The *y*-axis shows the four clusters derived by Lachesis. Due to the presence of strong chromatin domains on chromosome I, Lachesis was unable to merge this chromosome into a single cluster and required an input of *N* = 4.

We created a simulated draft *de novo* metagenome assembly for M-3D by splitting the reference genomes of the 18 species into 10-kb contigs. We also experimentally generated a Hi-C sequencing library for the M-3D sample (Table 2), aligned these reads to the simulated contigs of the draft assembly, and clustered the contigs using Hi-C link frequencies (Figure 3A). Our algorithm predicted the presence of 18 distinct clusters, consistent with the actual content of the simulated draft assembly and experimental Hi-C data (Figure S4). It clustered 89.1% of the simulated contigs into these 18 clusters; of the unclustered contigs, 85.8% contained no *Hin*dIII restriction sites and thus are not expected to produce a Hi-C signal in this experiment. The 18 clusters clearly matched the 18 species in the sample, with 99.6% of contigs clustered correctly (Figure 3B and Figure S9). The clusters corresponding to archaeal and bacterial species had a particularly high accuracy rate of 99.87%. Bootstrapping tests confirmed the robustness of our method (Table S3). Thus, our approach can simultaneously deconvolute the genomes of microbes belonging to all three domains of life, making it applicable to real and complex microbial communities.

Finally, we sought to use Hi-C to scaffold the genomic content of prokaryotic species from clustered contigs. Consistent with previous findings (Umbarger *et al.* 2011), we observed in the M-3D sample that both bacterial and archaeal genomes contain a substantially weaker signal of genomic proximity in Hi-C data than do eukaryotic genomes (Figure S10). This suggests that in prokaryotic species, in sharp contrast with eukaryotic species, Hi-C is not very useful for ordering or orienting genomic content within chromosomes. However, Lachesis’ clustering algorithm can still be used to deconvolute chromosomes, including plasmids, inside prokaryotic cells. We applied this algorithm to the genome of *Vibrio fischeri* ES114, a bacterial strain present in M-3D that contains two chromosomes and one plasmid, pES100 (Figure 3C). The chromosomal architecture of *V. fischeri* prevented a complete merging of its chromosome I, but chromosome II and pES100 both formed distinct clusters (Figure 3D). Thus, MetaPhase and Lachesis are capable of using Hi-C signal not only to deconvolute prokaryotic genomes but also to separate plasmid-derived sequence from chromosomal sequence within clusters corresponding to individual species.

## Discussion

Here, we demonstrate that contact probability maps such as those generated by Hi-C enable the deconvolution of shotgun metagenome assemblies and the reconstruction of individual genomes from mixed cell populations. Using only a single Hi-C library taken from a metagenome sample, we exploit two different signals inherent to Hi-C read pairing: the intracellularity of each pair, which enables species-level deconvolution, and the correlation of Hi-C linkage with chromosomal distance, which enables scaffolding of the *de novo* assemblies of at least eukaryotic species, as we have previously shown (Burton *et al.* 2013). All of the sequencing libraries used here were generated by *in vitro* methods and were sequenced on a single cost-effective sequencing platform.

The MetaPhase method is straightforward enough to be applicable to any metagenome sample from which a sufficient number of intact microbial cells can be isolated (10^5^–10^8^). Furthermore, this approach can be applied to microbial communities containing both prokaryotes and eukaryotes. The application of MetaPhase to diverse microbial communities may permit the discovery and genome assembly of many unculturable and currently unknown microbial species. Additionally, the use of the intracluster enrichment metric (Figure S4) permits a rough estimate of the species diversity within a draft metagenome assembly, a useful piece of information that is not easily measured. However, as with all shotgun metagenomic sequencing, low-abundance species—such as *P. pastoris* in our M-Y sample—will remain challenging to assemble into contigs without very deep sequencing. Additionally, in samples containing species such as dinoflagellates with unusually large genomes (Moustafa *et al.* 2010), even deeper sequencing of both shotgun and Hi-C libraries may be necessary.

We note that as MetaPhase delineates genomic content corresponding to individual microbial species, it also informs the chromosome and plasmid structure of these genomes and, in the case of eukaryotic species, it is capable of facilitating high-contiguity draft genome assemblies. Thus, it makes new species immediately amenable to phylogenetic and functional analysis while concomitantly increasing the power of existing genome databases to classify metagenomic reads via non-*de novo* methods. This method need not be limited to metagenome samples, because any complex cell mixture may be deconvoluted into individual genomes assuming enough genomic diversity is present that reads can be accurately mapped.

### Software availability

The computational portions of the MetaPhase method consist of software written in C++ using Boost (http://www.boost.org) with auxiliary scripts written in Perl and bash. It runs in a Unix environment. The source code has been uploaded to GitHub and is freely available for public download at https://github.com/shendurelab/MetaPhase.

## Supplementary Material

Supporting Information
