# Supplementary material for: Species-Level Deconvolution of Metagenome Assemblies with Hi-C–Based Contact Probability Maps
Source: G3 (Bethesda). 2014 May 22;4(7):1339–46. doi: 10.1534/g3.114.011825 (PMC4455782; doi:10.1534/g3.114.011825)
Supplement: Supporting Information [file supp_4_7_1339__index.html]

Species-Level Deconvolution of Metagenome Assemblies with Hi-C–Based Contact Probability Maps — Supporting Information 

# Species-Level Deconvolution of Metagenome Assemblies with Hi-C–Based Contact Probability Maps

## Supporting Information for Burton *et al.*, 2014

**Files in this Data Supplement:**

- Supporting Information - Tables S1-S3 and Figures S1-S10 (PDF, 1 MB)
- Table S1 - M-Y species list and abundances in sample. (PDF, 118 KB)
- Table S2 - M-3D species list and abundances in sample. (PDF, 119 KB)
- Table S3 - Clustering results on bootstrapped Hi-C link datasets. (PDF, 116 KB)
- Figure S1 - M-Y species phylogeny. (PDF, 178 KB)
- Figure S2 - M-Y sequence divergences between species. (PDF, 178 KB)
- Figure S3 - Coverage of M-Y reference genomes by draft metagenome assembly. (PDF, 212 KB)
- Figure S4 - Intra-cluster link enrichment as a function of cluster number in M-Y and M-3D. (PDF, 181 KB)
- Figure S5 - Heatmap of non-unique reference alignments of contigs in each M-Y cluster. (PDF, 193 KB)
- Figure S6 - Differential Hi-C efficiency rates by species for the M-Y sample. (PDF, 185 KB)
- Figure S7 - Accuracy of Lachesis assembly of *Scheffersomyces stipitis*. (PDF, 199 KB)
- Figure S8 - M-3D species phylogeny. (PDF, 179 KB)
- Figure S9 - Heatmap of non-unique reference alignments of contigs in each M-3D cluster. (PDF, 347 KB)
- Figure S10 - Heatmaps of M-3D Hi-C links aligned to prokaryotic reference genomes. (PDF, 334 KB)
