# Supplementary material for: Species-Level Deconvolution of Metagenome Assemblies with Hi-C–Based Contact Probability Maps
Source: G3 (Bethesda). 2014 May 22;4(7):1339–46. doi: 10.1534/g3.114.011825 (PMC4455782; doi:10.1534/g3.114.011825)
Supplement: Supporting Information [file supp_g3.114.011825_011825SI.pdf]

G3 · Genes | Genomes | Genetics

Species-Level Deconvolution of Metagenome Assemblies with Hi-C-Based Contact Probability Maps

Joshua N. Burton*, Ivan Liachko*, Maitreya J. Dunham, Jay Shendure

Department of Genome Sciences, University of Washington, Seattle, WA 98105.

Corresponding authors: Maitreya J. Dunham and Jay Shendure, University of Washington, Foege Building S-250, Box 355065

3720 15th Ave NE, Seattle, WA 98105-5065. (206) 685-3720, maitreya@uw.edu, shendure@uw.edu.

* These authors contributed equally to this work.

DOI: 10.1534/g3.114.011825

Table S1 M-Y species list and abundances in sample

Genus	Species	In sample				Reference			
		Strain	Source	Ploidy	Optical density	Strain	Size (Mb)	Download source	Finished?
Saccharomyces	*cerevisiae*	FY4H	M. Dunham	1	0.079088	FY	12.2	downloads.yeastgenome.org	Yes
Saccharomyces	*cerevisiae*	CEN.PK	P. Kotter	1	0.071645	CEN.PK	11.5	downloads.yeastgenome.org	No
Saccharomyces	*cerevisiae*	RM11-1A	L. Kruglyak	1	0.084903	RM11-1A	11.7	www.broadinstitute.org	Yes
Saccharomyces	*cerevisiae*	SK1	A. Deutschbauer	2	0.075366	SK1	11.9	cbio.mskcc.org/public/SK1_MvO/	Yes
Saccharomyces	*paradoxus*	YDG613	D. Greig	2	0.076762		11.7	saccharomycessensustricto.org	Yes
Saccharomyces	*mikatae*	FM356	M. Johnston	2	0.08188	IFO 1815	11.5	saccharomycessensustricto.org	Yes
Saccharomyces	*kudriavzevii*	FM527	M. Johnston	2	0.008141	IFO 1802	11.3	saccharomycessensustricto.org	Yes
Saccharomyces	*bayanus* var. *uvarum*	YZB5-113	Y. Zhang	1	0.055827	CBS 7001	11.5	saccharomycessensustricto.org	Yes
Naumovozyma (*Saccharomyces*)	*castellii*	4310	D. Bartel	1	0.082577	NRRL Y-12630	11.2	downloads.yeastgenome.org	No
Lachancea	*waltii*	Kwaltii ura3	B. Brewer	1	0.086067	NRRL Y-8285	10.2	fangman-brewer-gbrowse.gs.washington.edu	Mostly
Lachancea (*Saccharomyces*)	*kluyveri*	FM628	M. Johnston	1	0.096534	CBS 3082	11.3	genolevures.org/sakl.html	Yes
Kluyveromyces	*lactis*	MW98-8C	C. Newlon	1	0.055827	NRRL Y-1140	10.7	genolevures.org/klla.html	Yes
Kluyveromyces	*wickerhamii*	Y-8286	USDA/ARS	1	0.062805	UCD 54-210	9.81	www.ncbi.nlm.nih.gov	No
Ashbya (*Eremothecium*)	*gossypii*	WT	S. Jaspersen	1	Can't measure	ATCC 10895	8.74	genolevures.org/ergo.html	Yes
Scheffersomyces (*Pichia*)	*stipitis*	Y-11545	USDA/ARS	1	0.080251	CBS 6054	15.4	www.ncbi.nlm.nih.gov	Yes
Pichia (*Komagataella*)	*pastoris*	JC308	J. Cregg	1	0.002326	GS115	9.21	www.ncbi.nlm.nih.gov	Yes

Table S2 M-3D species list and abundances in sample

Domain	Genus	Species	In sample			Reference			
			Strain	Source	Optical density	Strain	Size (Mb)	Download source	Finished?
Eukaryota	*Saccharomyces*	*cerevisiae*	FY4H	M. Dunham	1.02	FY	12.2	downloads.yeastgenome.org	Yes
Eukaryota	*Zygosaccharomyces*	*rouxii*	Y-229	USDA/ARS	0.66	CBS 732	9.76	genolevures.org/zyro.html	Yes
Eukaryota	*Lachancea* (*Kluyveromyces*)	*thermotolerans*	Y-8284	USDA/ARS	0.98	CBS 6340	9.39	genolevures.org/klth.html	Yes
Eukaryota	*Kluyveromyces*	*aestuarii*	YB-4510	USDA/ARS	1.04	ATCC 18862	9.91	www.ncbi.nlm.nih.gov	No
Eukaryota	*Hansenula* (*Ogataea*)	*polymorpha*	Y-5445	USDA/ARS	1.21	DL-1	8.86	www.ncbi.nlm.nih.gov	Yes
Eukaryota	*Pichia* (*Komagataella*)	*pastoris*	JC 308	J. Cregg	0.61	GS115	9.22	www.ncbi.nlm.nih.gov	Yes
Eukaryota	*Schizosaccharomyces*	*pombe*	YFS 103	N. Rhind	0.52	ASM294	12.6	www.pombase.org	Yes
Eukaryota	*Schizosaccharomyces*	*japonicus*	YFS 760	N. Rhind	0.19	yFS275	11.7	www.broadinstitute.org	No
Archaea	*Methanococcus*	*maripaludis*	S2	J. Leigh	~0.1	S2	1.67	www.ncbi.nlm.nih.gov	Yes
Bacteria	*Escherichia*	*coli*	AG 111	H. Merrikh	0.26	K-12	4.69	www.ncbi.nlm.nih.gov	Yes
Bacteria	*Vibrio* (*Aliivibrio*)	*fischeri*	ES114	P. Greenberg	0.25	ES114	4.27	www.ncbi.nlm.nih.gov	Yes
Bacteria	*Pseudomonas*	*fluorescens*	Pf-5	C. Harwood	0.4	Pf0-1	6.44	www.ncbi.nlm.nih.gov	Yes
Bacteria	*Acinetobacter*	*baylyi*	ADP1	C. Harwood	0.12	ADP1	3.60	www.ncbi.nlm.nih.gov	Yes
Bacteria	*Burkholderia*	*thailandensis*	E264	C. Harwood	0.6	E264	6.72	www.ncbi.nlm.nih.gov	Yes
Bacteria	*Agrobacterium*	*tumefaciens*	P4	C. Queitsch	0.37	P4	6.33	www.ncbi.nlm.nih.gov	Mostly
Bacteria	*Rhodopseudomonas*	*palustris*	CGA009	C. Harwood	0.32	CGA 009	5.47	www.ncbi.nlm.nih.gov	Yes
Bacteria	*Flavobacterium*	*johnsoniae*	UW 101	C. Harwood	0.55	UW 101	6.10	www.ncbi.nlm.nih.gov	Yes
Bacteria	*Bacillus*	*subtilis*	HM1/168	H. Merrikh	0.35	168	4.22	www.ncbi.nlm.nih.gov	Yes

Table S3 Clustering results on bootstrapped Hi-C link datasets

	Sequence clustered	% clustered	Seq misclustered	% misclustered
**M-Y (total sequence length = 135206617)**				
	Sequence clustered	% clustered	Seq misclustered	% misclustered
**Main result**	111112059	82.18%	922932	0.83%
**Bootstrap 1**	111136126	82.20%	4146798	3.73%
**Bootstrap 2**	111102736	82.17%	4500167	4.05%
**Bootstrap 3**	111106339	82.18%	4655083	4.19%
**Bootstrap 4**	111101816	82.17%	4389061	3.95%
**Bootstrap 5**	111106542	82.18%	4425448	3.98%
**Bootstrap 6**	111158559	82.21%	4356095	3.92%
**Bootstrap 7**	111089140	82.16%	4173561	3.76%
**Bootstrap 8**	110777343	81.93%	1294345	1.17%
**M-PE (total sequence length = 133169811)**				
	Sequence clustered	% clustered	Seq misclustered	% misclustered
**Main result**	118841530	89.24%	461626	0.39%
**Bootstrap 1**	117677687	88.37%	1748183	1.49%
**Bootstrap 2**	117818421	88.47%	737953	0.63%
**Bootstrap 3**	117636660	88.34%	1834184	1.56%
**Bootstrap 4**	117604654	88.31%	497732	0.42%
**Bootstrap 5**	117695244	88.38%	509778	0.43%
**Bootstrap 6**	117566728	88.28%	1600895	1.36%
**Bootstrap 7**	117679867	88.37%	1870031	1.59%
**Bootstrap 8**	117760573	88.43%	1748183	1.48%

We ran the MetaPhase clustering algorithm on the M-Y and M-PE datasets, producing the results given in the main Results section. We also re-ran the clustering algorithm in each of these cases and applied randomized bootstrapping (that is, re-sampling with replacement of N data points) to the Hi-C link data. Shown are the results of eight bootstrapping runs for each sample.

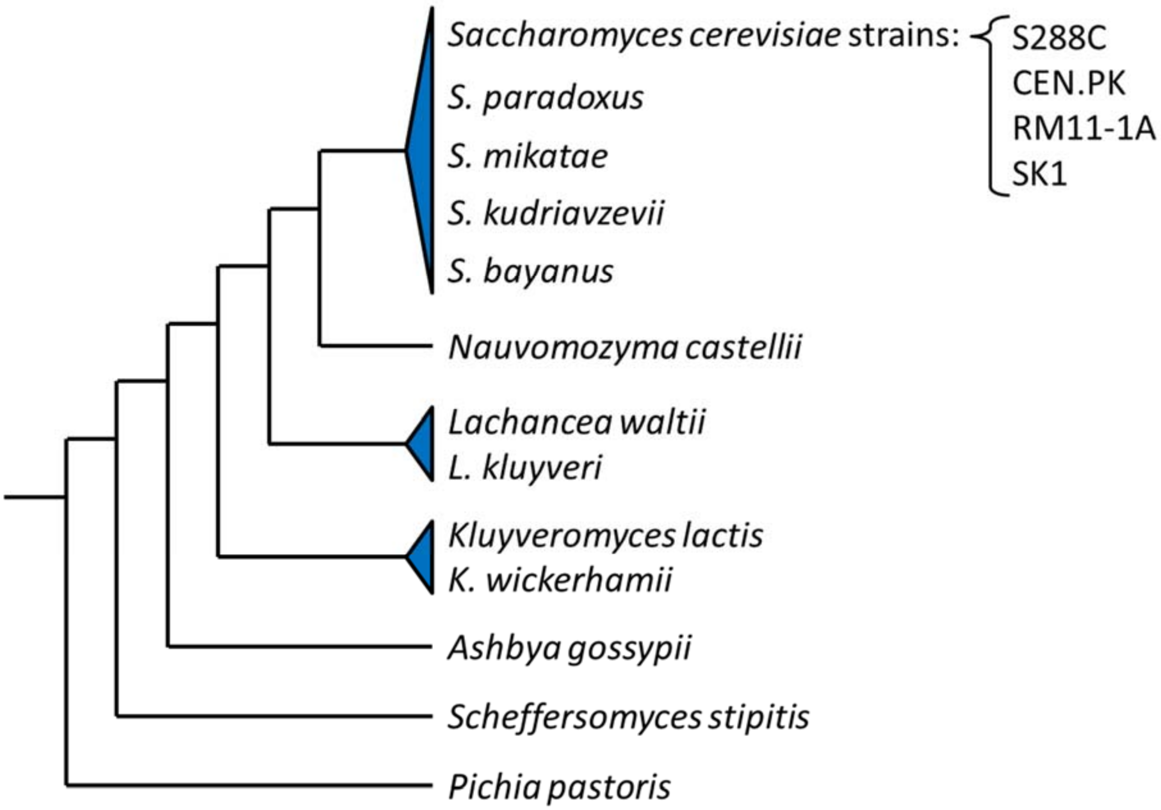

Figure S1 M-Y species phylogeny. Phylogenetic tree of the 16 Ascomycetes yeast strains used in the M-Y sample (Table S1).

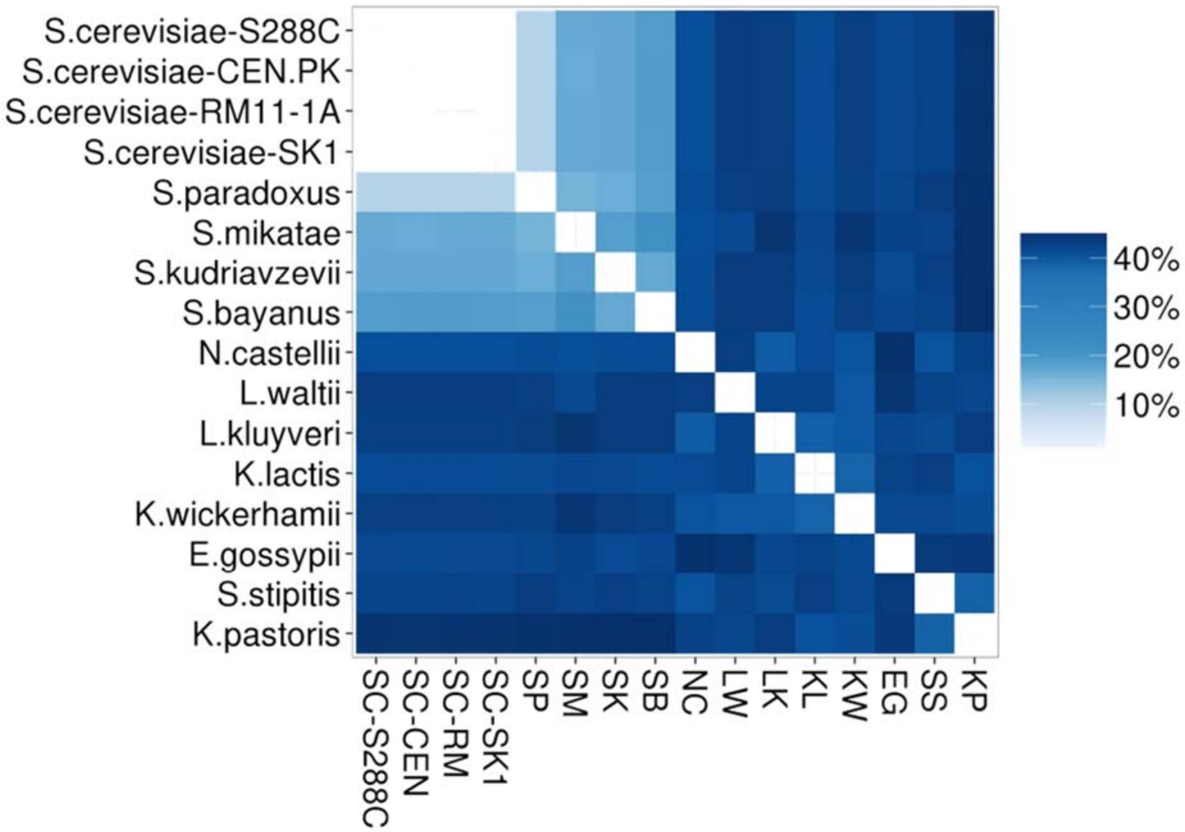

Figure S2 M-Y sequence divergences between species. Divergence rates were calculated as follows: First, a set of essential ORFs in the *Saccharomyces cerevisiae* genome was downloaded from the Yeast Deletion Website. For each essential ORF, orthologous sequences in every other species were found via BLASTn alignment (Altschul *et al.* 1990), and these sequences were all aligned together using Clustal Omega (Sievers *et al.* 2011). Pairwise divergences were calculated by counting the frequency of mismatches among aligned base pairs in the Clustal Omega alignments. This analysis was repeated using essential ORFs from *K. lactis* instead of *S. cerevisiae*, with very similar results (data not shown).

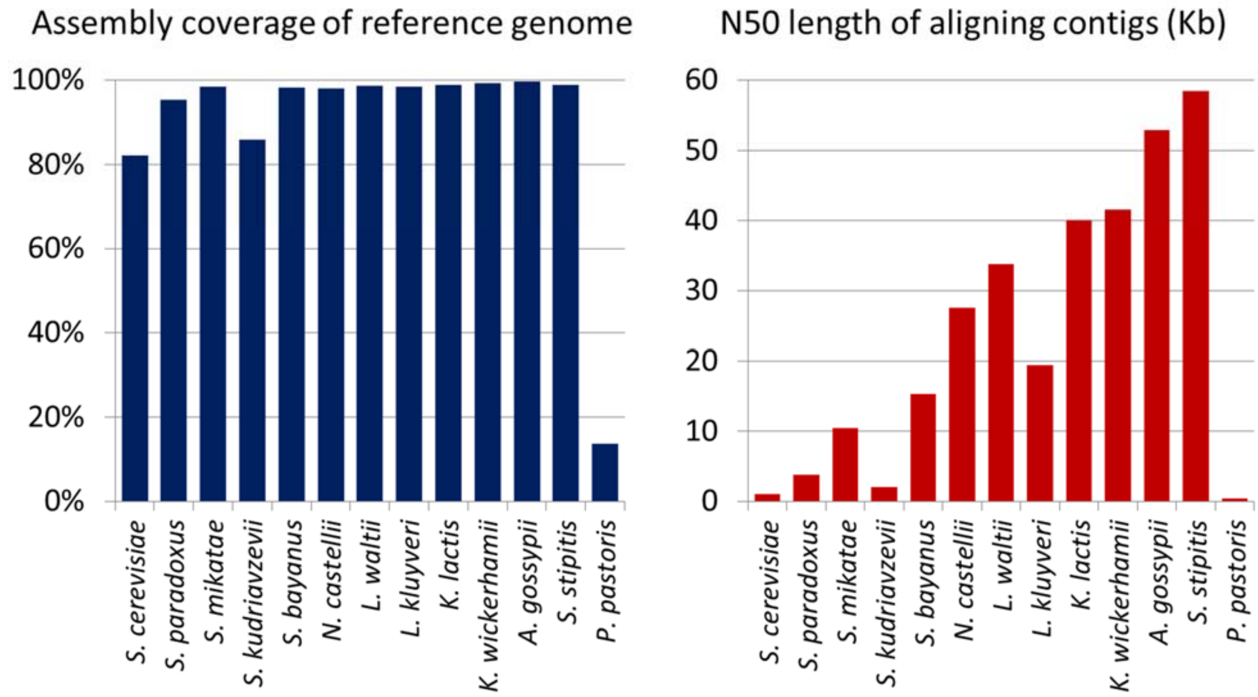

Figure S3 Coverage of M-Y reference genomes by draft metagenome assembly. Contigs from the M-Y draft metagenome assembly were aligned to the reference genomes of each species with BLASTn (Altschul *et al.* 1990) using the following parameters: `-perc_identity 95 -evalue 1e-30 -word_size 50`. The restrictiveness of these parameters ensured that all alignments generated were greater than 70 bp. Left: The fraction of each reference genome covered by BLASTn alignments. Right: For each reference genome, the N50 length of draft contigs aligning to that genome.

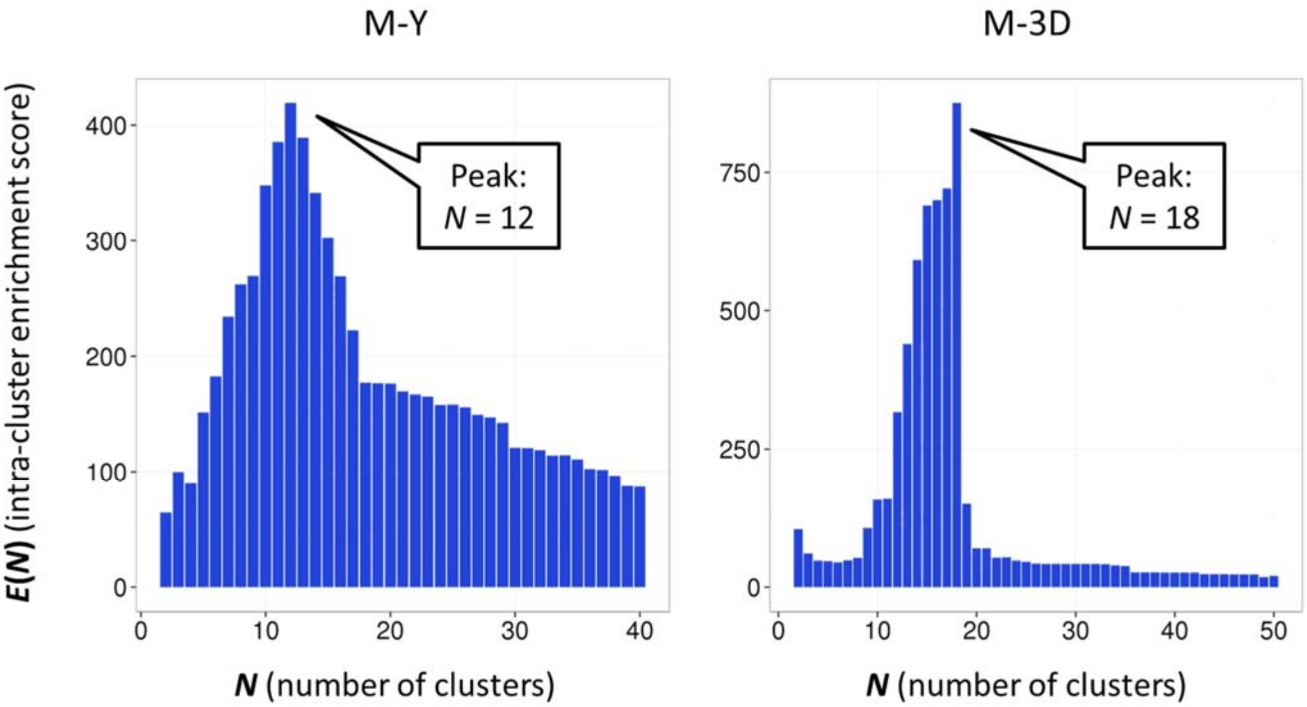

Figure S4 Intra-cluster link enrichment as a function of cluster number in M-Y and M-3D. We ran the hierarchical agglomerative clustering algorithm on the M-Y and M-3D datasets. In this algorithm, the number of clusters gradually decreases as clusters are merged together; to generate this data, we continued clustering all the way down to $N = 1$. Shown is the metric E, or intra-cluster link enrichment, at each value of N. Note that for both M-Y and M-3D the maximum value of $E(N)$ occurs when N is equal to the true number of distinct species present in the draft assembly.

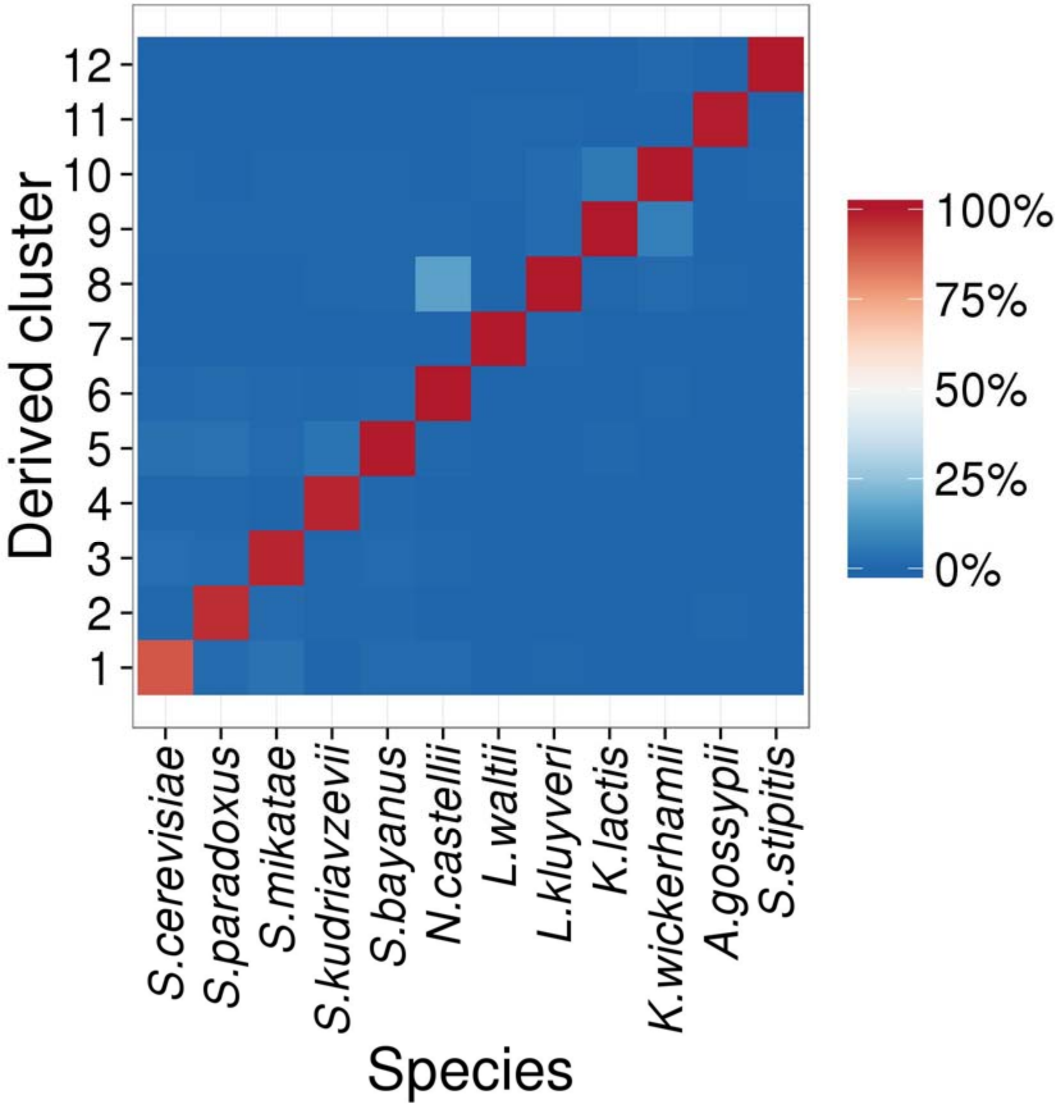

Figure S5 Heatmap of non-unique reference alignments of contigs in each M-Y cluster. This is identical to Figure 2B, except that all contig alignments to all genomes are shown here, whereas in Figure 2B only contigs that align uniquely to a single reference genome are shown.

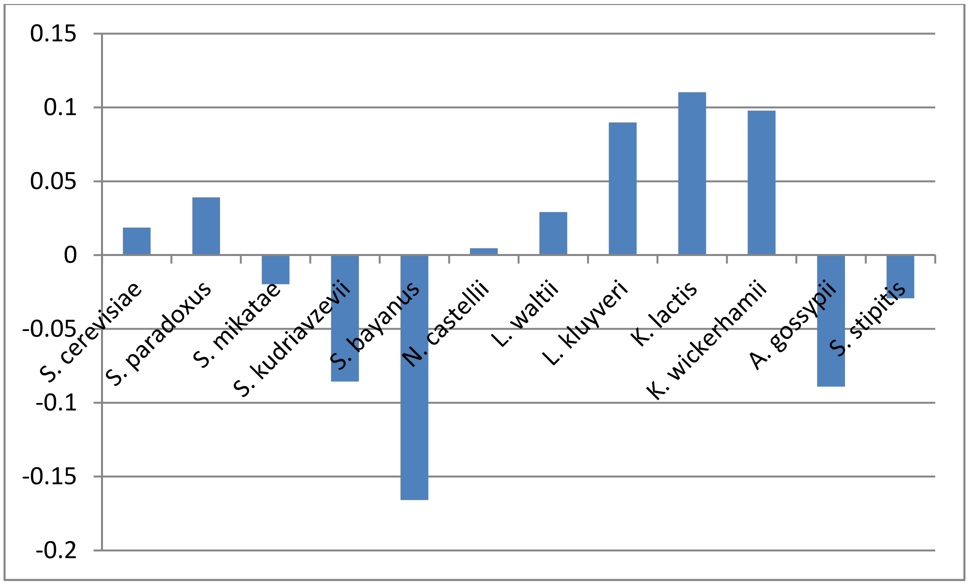

Figure S6 Differential Hi-C efficiency rates by species for the M-Y sample. For each species, the Hi-C efficiency rate was calculated as $E_{species} = \frac{f_{species}(\text{Hi-C})}{f_{species}(\text{shotgun})}$, where $f_{species}$(library) is the fraction of reads from a sequencing library that align to the given species' reference genome. These efficiency rates were log-scaled and then normalized to create an average of 0 over all species.

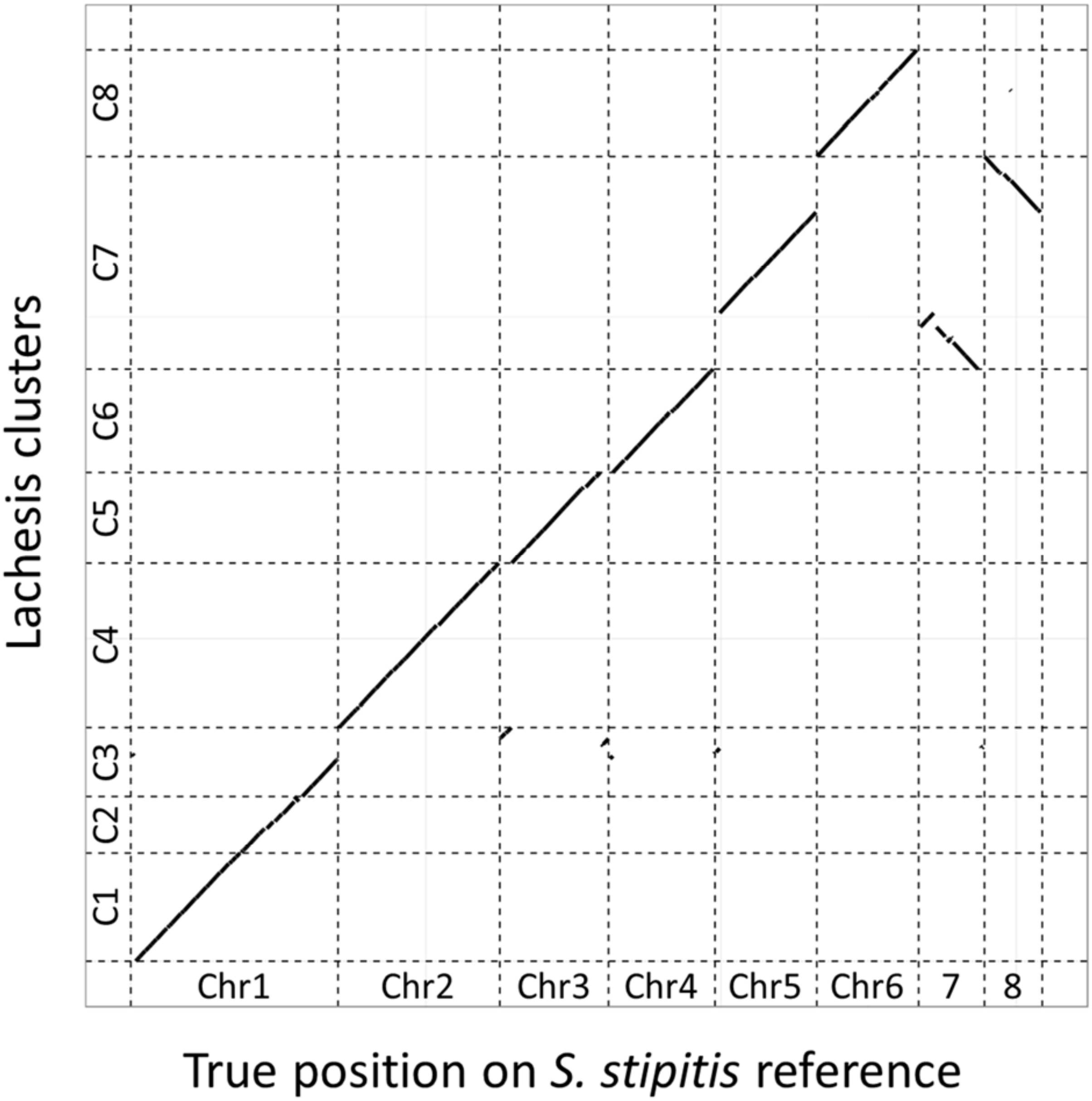

Figure S7 Accuracy of Lachesis assembly of *Scheffersomyces stipitis*. The contigs in the MetaPhase cluster corresponding to *S. stipitis* were clustered, ordered, and oriented with Lachesis (Burton *et al.* 2013) (Figure 2C). Shown here is a validation of the Lachesis assembly. Every contig that is placed by Lachesis and which aligns to the *S. stipitis* reference genome is shown. *x*-axis: the contig's true position in the *S. stipitis* reference. *y*-axis: the contig's placement in the Lachesis assembly (note that both the order of the clusters on the *y*-axis and the overall orientation of each cluster are arbitrary; they are chosen here for visual clarity and are not the same as in Figure 2C.)

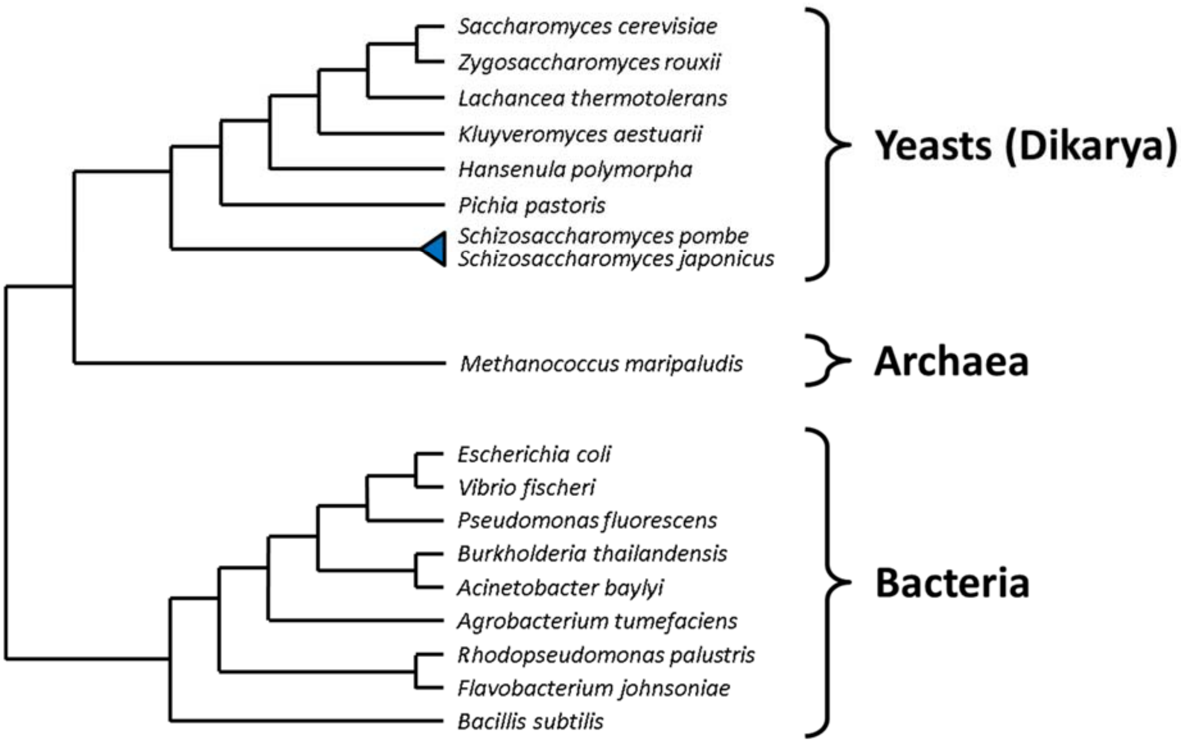

Figure S8 M-3D species phylogeny. Phylogenetic tree of the 18 yeast, archaeal, and bacterial strains used in the M-3D sample (Table S2).

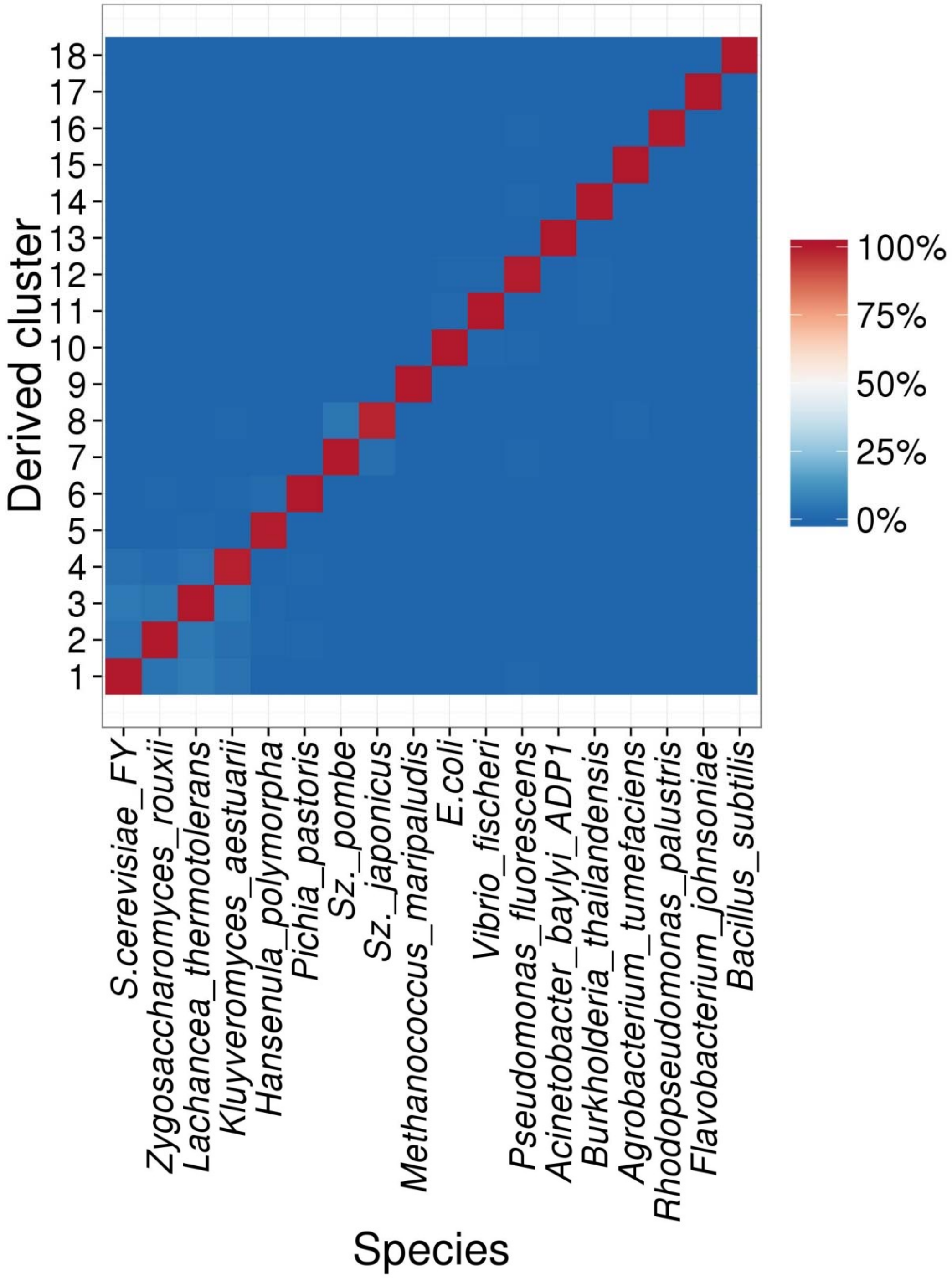

Figure S9 Heatmap of non-unique reference alignments of contigs in each M-3D cluster. This is identical to Figure 3B, except that all contig alignments to all genomes are shown here, whereas in Figure 3B only contigs that align uniquely to a single reference genome are shown.

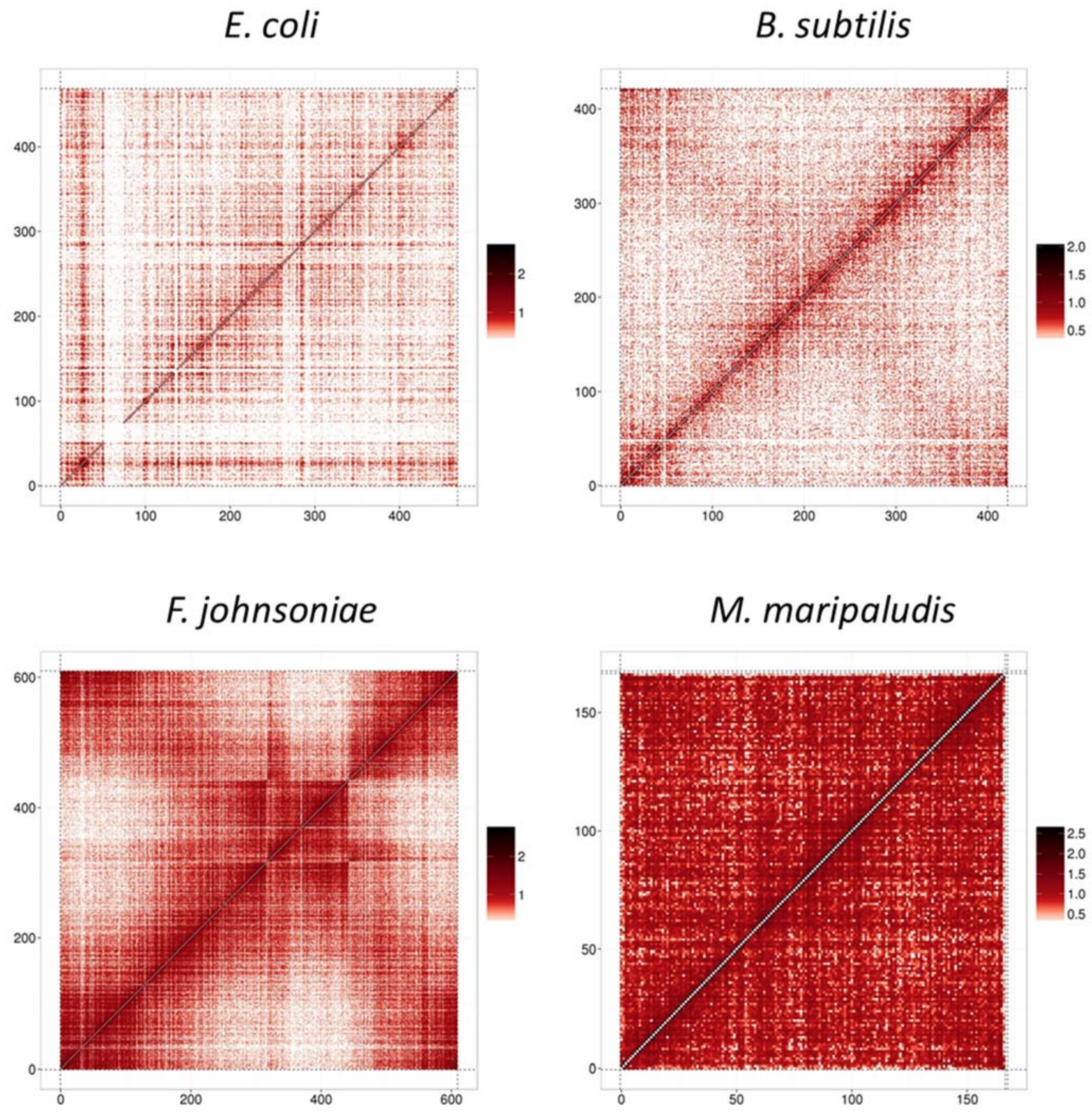

Figure S10 Heatmaps of M-3D Hi-C links aligned to prokaryotic reference genomes. Reads from the M-3D *Hin*dIII non-resuspended library (Table S3) were aligned to the draft assemblies of four prokaryotic species present in the M-3D sample. Each heatmap has a resolution of 10 Kb, and the legend indicates the $\log_{10}$ of link density.
