# Supplementary material for: Species-Level Deconvolution of Metagenome Assemblies with Hi-C–Based Contact Probability Maps
Source: G3 (Bethesda). 2014 May 22;4(7):1339–46. doi: 10.1534/g3.114.011825 (PMC4455782; doi:10.1534/g3.114.011825)
Supplement: Supporting Information [file supp_g3.114.011825_TableS1.pdf]

Table S1 M-Y species list and abundances in sample

Genus	Species	In sample				Reference			
		Strain	Source	Ploidy	Optical density	Strain	Size (Mb)	Download source	Finished?
Saccharomyces	*cerevisiae*	FY4H	M. Dunham	1	0.079088	FY	12.2	downloads.yeastgenome.org	Yes
Saccharomyces	*cerevisiae*	CEN.PK	P. Kotter	1	0.071645	CEN.PK	11.5	downloads.yeastgenome.org	No
Saccharomyces	*cerevisiae*	RM11-1A	L. Kruglyak	1	0.084903	RM11-1A	11.7	www.broadinstitute.org	Yes
Saccharomyces	*cerevisiae*	SK1	A. Deutschbauer	2	0.075366	SK1	11.9	cbio.mskcc.org/public/SK1_MvO/	Yes
Saccharomyces	*paradoxus*	YDG613	D. Greig	2	0.076762		11.7	saccharomycessensustricto.org	Yes
Saccharomyces	*mikatae*	FM356	M. Johnston	2	0.08188	IFO 1815	11.5	saccharomycessensustricto.org	Yes
Saccharomyces	*kudriavzevii*	FM527	M. Johnston	2	0.008141	IFO 1802	11.3	saccharomycessensustricto.org	Yes
Saccharomyces	*bayanus* var. *uvarum*	YZB5-113	Y. Zhang	1	0.055827	CBS 7001	11.5	saccharomycessensustricto.org	Yes
Naumovozyma (*Saccharomyces*)	*castellii*	4310	D. Bartel	1	0.082577	NRRL Y-12630	11.2	downloads.yeastgenome.org	No
Lachancea	*waltii*	Kwaltii ura3	B. Brewer	1	0.086067	NRRL Y-8285	10.2	fangman-brewer-gbrowse.gs.washington.edu	Mostly
Lachancea (*Saccharomyces*)	*kluyveri*	FM628	M. Johnston	1	0.096534	CBS 3082	11.3	genolevures.org/sakl.html	Yes
Kluyveromyces	*lactis*	MW98-8C	C. Newlon	1	0.055827	NRRL Y-1140	10.7	genolevures.org/klla.html	Yes
Kluyveromyces	*wickerhamii*	Y-8286	USDA/ARS	1	0.062805	UCD 54-210	9.81	www.ncbi.nlm.nih.gov	No
Ashbya (*Eremothecium*)	*gossypii*	WT	S. Jaspersen	1	Can't measure	ATCC 10895	8.74	genolevures.org/ergo.html	Yes
Scheffersomyces (*Pichia*)	*stipitis*	Y-11545	USDA/ARS	1	0.080251	CBS 6054	15.4	www.ncbi.nlm.nih.gov	Yes
Pichia (*Komagataella*)	*pastoris*	JC308	J. Cregg	1	0.002326	GS115	9.21	www.ncbi.nlm.nih.gov	Yes
