# Supplementary material for: Species-Level Deconvolution of Metagenome Assemblies with Hi-C–Based Contact Probability Maps
Source: G3 (Bethesda). 2014 May 22;4(7):1339–46. doi: 10.1534/g3.114.011825 (PMC4455782; doi:10.1534/g3.114.011825)
Supplement: Supporting Information [file supp_g3.114.011825_TableS2.pdf]

Table S2 M-3D species list and abundances in sample

Domain	Genus	Species	In sample			Reference			
			**Strain**	**Source**	**Optical density**	**Strain**	**Size (Mb)**	**Download source**	**Finished?**
Eukaryota	*Saccharomyces*	*cerevisiae*	FY4H	M. Dunham	1.02	FY	12.2	downloads.yeastgenome.org	Yes
Eukaryota	*Zygosaccharomyces*	*rouxii*	Y-229	USDA/ARS	0.66	CBS 732	9.76	genolevures.org/zyro.html	Yes
Eukaryota	*Lachancea* (*Kluyveromyces*)	*thermotolerans*	Y-8284	USDA/ARS	0.98	CBS 6340	9.39	genolevures.org/klth.html	Yes
Eukaryota	*Kluyveromyces*	*aestuarii*	YB-4510	USDA/ARS	1.04	ATCC 18862	9.91	www.ncbi.nlm.nih.gov	No
Eukaryota	*Hansenula* (*Ogataea*)	*polymorpha*	Y-5445	USDA/ARS	1.21	DL-1	8.86	www.ncbi.nlm.nih.gov	Yes
Eukaryota	*Pichia* (*Komagataella*)	*pastoris*	JC 308	J. Cregg	0.61	GS115	9.22	www.ncbi.nlm.nih.gov	Yes
Eukaryota	*Schizosaccharomyces*	*pombe*	YFS 103	N. Rhind	0.52	ASM294	12.6	www.pombase.org	Yes
Eukaryota	*Schizosaccharomyces*	*japonicus*	YFS 760	N. Rhind	0.19	yFS275	11.7	www.broadinstitute.org	No
Archaea	*Methanococcus*	*maripaludis*	S2	J. Leigh	~0.1	S2	1.67	www.ncbi.nlm.nih.gov	Yes
Bacteria	*Escherichia*	*coli*	AG 111	H. Merrikh	0.26	K-12	4.69	www.ncbi.nlm.nih.gov	Yes
Bacteria	*Vibrio* (*Aliivibrio*)	*fischeri*	ES114	P. Greenberg	0.25	ES114	4.27	www.ncbi.nlm.nih.gov	Yes
Bacteria	*Pseudomonas*	*fluorescens*	Pf-5	C. Harwood	0.4	Pf0-1	6.44	www.ncbi.nlm.nih.gov	Yes
Bacteria	*Acinetobacter*	*baylyi*	ADP1	C. Harwood	0.12	ADP1	3.60	www.ncbi.nlm.nih.gov	Yes
Bacteria	*Burkholderia*	*thailandensis*	E264	C. Harwood	0.6	E264	6.72	www.ncbi.nlm.nih.gov	Yes
Bacteria	*Agrobacterium*	*tumefaciens*	P4	C. Queitsch	0.37	P4	6.33	www.ncbi.nlm.nih.gov	Mostly
Bacteria	*Rhodopseudomonas*	*palustris*	CGA009	C. Harwood	0.32	CGA 009	5.47	www.ncbi.nlm.nih.gov	Yes
Bacteria	*Flavobacterium*	*johnsoniae*	UW 101	C. Harwood	0.55	UW 101	6.10	www.ncbi.nlm.nih.gov	Yes
Bacteria	*Bacillus*	*subtilis*	HM1/168	H. Merrikh	0.35	168	4.22	www.ncbi.nlm.nih.gov	Yes
