# Supplementary material for: Species-Level Deconvolution of Metagenome Assemblies with Hi-C–Based Contact Probability Maps
Source: G3 (Bethesda). 2014 May 22;4(7):1339–46. doi: 10.1534/g3.114.011825 (PMC4455782; doi:10.1534/g3.114.011825)
Supplement: Supporting Information [file supp_g3.114.011825_TableS3.pdf]

Table S3 Clustering results on bootstrapped Hi-C link datasets

	Sequence clustered	% clustered	Seq misclustered	% misclustered
**M-Y (total sequence length = 135206617)**				
	Sequence clustered	% clustered	Seq misclustered	% misclustered
**Main result**	111112059	82.18%	922932	0.83%
**Bootstrap 1**	111136126	82.20%	4146798	3.73%
**Bootstrap 2**	111102736	82.17%	4500167	4.05%
**Bootstrap 3**	111106339	82.18%	4655083	4.19%
**Bootstrap 4**	111101816	82.17%	4389061	3.95%
**Bootstrap 5**	111106542	82.18%	4425448	3.98%
**Bootstrap 6**	111158559	82.21%	4356095	3.92%
**Bootstrap 7**	111089140	82.16%	4173561	3.76%
**Bootstrap 8**	110777343	81.93%	1294345	1.17%
**M-PE (total sequence length = 133169811)**				
	Sequence clustered	% clustered	Seq misclustered	% misclustered
**Main result**	118841530	89.24%	461626	0.39%
**Bootstrap 1**	117677687	88.37%	1748183	1.49%
**Bootstrap 2**	117818421	88.47%	737953	0.63%
**Bootstrap 3**	117636660	88.34%	1834184	1.56%
**Bootstrap 4**	117604654	88.31%	497732	0.42%
**Bootstrap 5**	117695244	88.38%	509778	0.43%
**Bootstrap 6**	117566728	88.28%	1600895	1.36%
**Bootstrap 7**	117679867	88.37%	1870031	1.59%
**Bootstrap 8**	117760573	88.43%	1748183	1.48%

We ran the MetaPhase clustering algorithm on the M-Y and M-PE datasets, producing the results given in the main Results section. We also re-ran the clustering algorithm in each of these cases and applied randomized bootstrapping (that is, re-sampling with replacement of N data points) to the Hi-C link data. Shown are the results of eight bootstrapping runs for each sample.
