# Supplementary material for: Species-Level Deconvolution of Metagenome Assemblies with Hi-C–Based Contact Probability Maps
Source: G3 (Bethesda). 2014 May 22;4(7):1339–46. doi: 10.1534/g3.114.011825 (PMC4455782; doi:10.1534/g3.114.011825)
Supplement: Supporting Information [file supp_g3.114.011825_FigureS1.pdf]

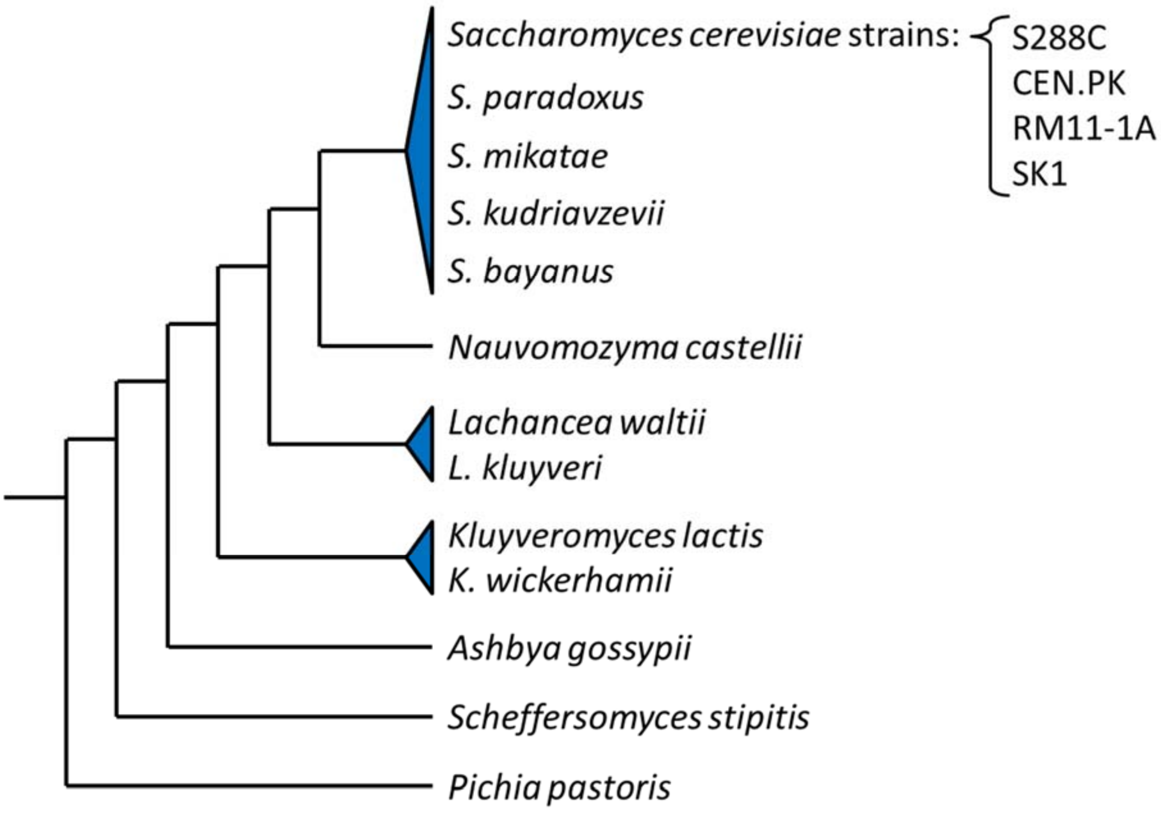

Figure S1 M-Y species phylogeny. Phylogenetic tree of the 16 Ascomycetes yeast strains used in the M-Y sample (Table S1).
