# Supplementary material for: Species-Level Deconvolution of Metagenome Assemblies with Hi-C–Based Contact Probability Maps
Source: G3 (Bethesda). 2014 May 22;4(7):1339–46. doi: 10.1534/g3.114.011825 (PMC4455782; doi:10.1534/g3.114.011825)
Supplement: Supporting Information [file supp_g3.114.011825_FigureS2.pdf]

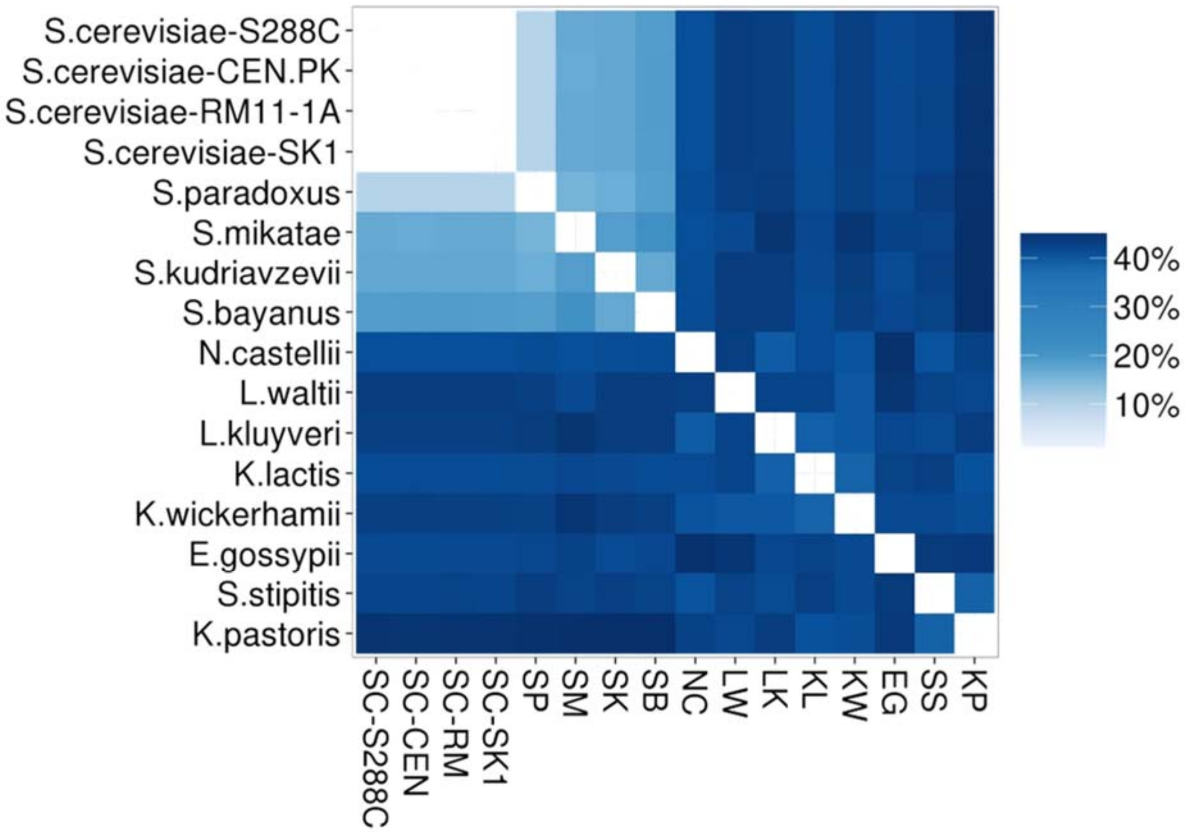

Figure S2 M-Y sequence divergences between species. Divergence rates were calculated as follows: First, a set of essential ORFs in the *Saccharomyces cerevisiae* genome was downloaded from the Yeast Deletion Website. For each essential ORF, orthologous sequences in every other species were found via BLASTn alignment (Altschul *et al.* 1990), and these sequences were all aligned together using Clustal Omega (Sievers *et al.* 2011). Pairwise divergences were calculated by counting the frequency of mismatches among aligned base pairs in the Clustal Omega alignments. This analysis was repeated using essential ORFs from *K. lactis* instead of *S. cerevisiae*, with very similar results (data not shown).
