# Supplementary material for: Species-Level Deconvolution of Metagenome Assemblies with Hi-C–Based Contact Probability Maps
Source: G3 (Bethesda). 2014 May 22;4(7):1339–46. doi: 10.1534/g3.114.011825 (PMC4455782; doi:10.1534/g3.114.011825)
Supplement: Supporting Information [file supp_g3.114.011825_FigureS3.pdf]

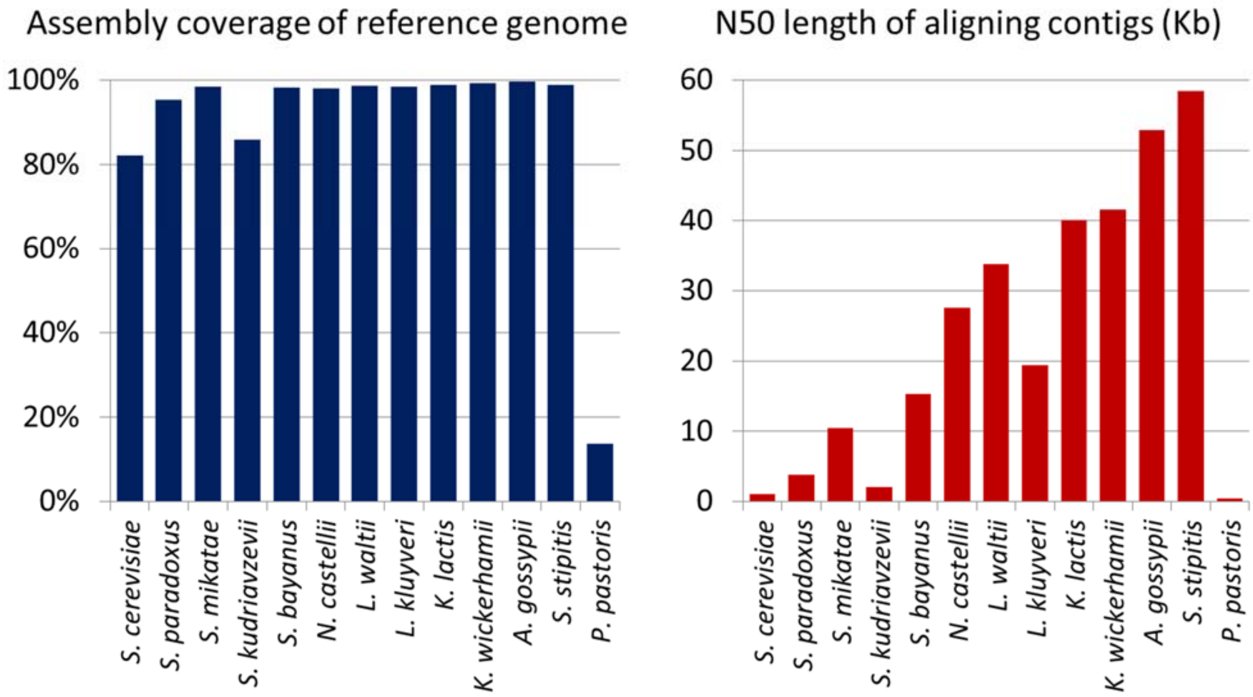

Figure S3 Coverage of M-Y reference genomes by draft metagenome assembly. Contigs from the M-Y draft metagenome assembly were aligned to the reference genomes of each species with BLASTn (Altschul *et al.* 1990) using the following parameters: `-perc_identity 95 -evalue 1e-30 -word_size 50`. The restrictiveness of these parameters ensured that all alignments generated were greater than 70 bp. Left: The fraction of each reference genome covered by BLASTn alignments. Right: For each reference genome, the N50 length of draft contigs aligning to that genome.
