# Supplementary material for: Species-Level Deconvolution of Metagenome Assemblies with Hi-C–Based Contact Probability Maps
Source: G3 (Bethesda). 2014 May 22;4(7):1339–46. doi: 10.1534/g3.114.011825 (PMC4455782; doi:10.1534/g3.114.011825)
Supplement: Supporting Information [file supp_g3.114.011825_FigureS4.pdf]

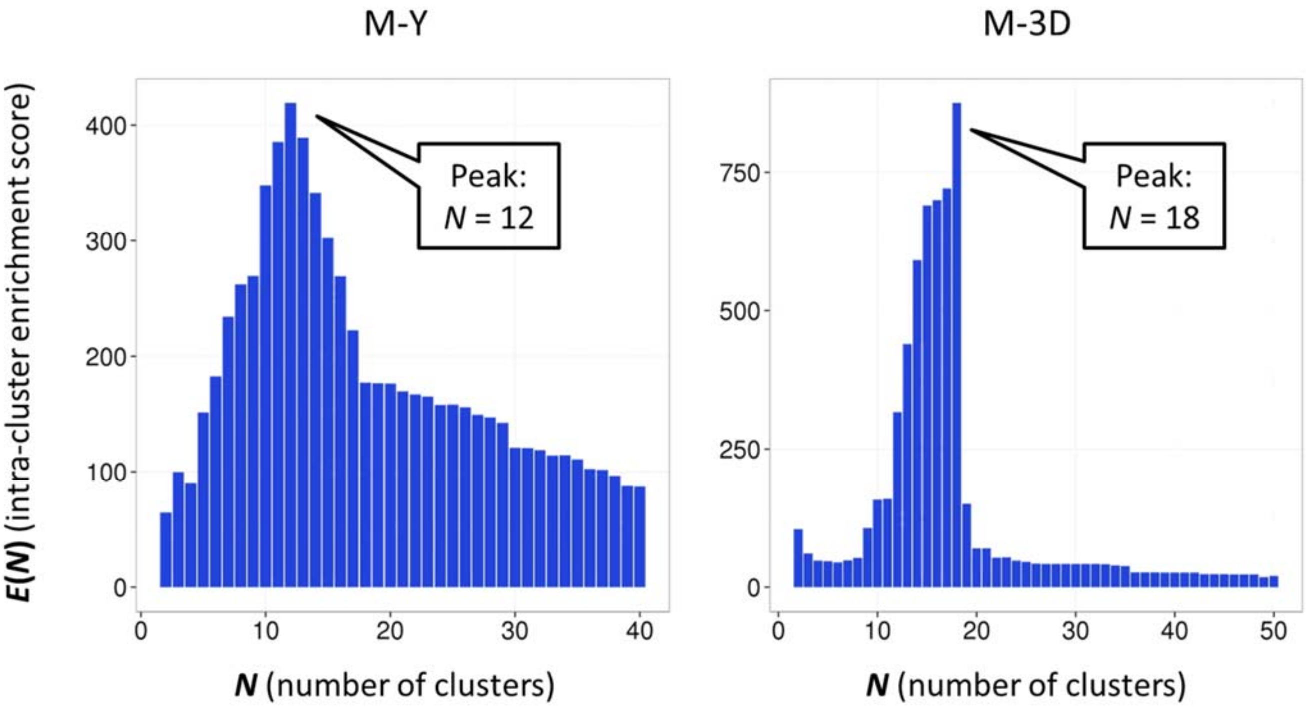

Figure S4 Intra-cluster link enrichment as a function of cluster number in M-Y and M-3D. We ran the hierarchical agglomerative clustering algorithm on the M-Y and M-3D datasets. In this algorithm, the number of clusters gradually decreases as clusters are merged together; to generate this data, we continued clustering all the way down to $N = 1$. Shown is the metric E, or intra-cluster link enrichment, at each value of N. Note that for both M-Y and M-3D the maximum value of $E(N)$ occurs when N is equal to the true number of distinct species present in the draft assembly.
