# Supplementary material for: Species-Level Deconvolution of Metagenome Assemblies with Hi-C–Based Contact Probability Maps
Source: G3 (Bethesda). 2014 May 22;4(7):1339–46. doi: 10.1534/g3.114.011825 (PMC4455782; doi:10.1534/g3.114.011825)
Supplement: Supporting Information [file supp_g3.114.011825_FigureS6.pdf]

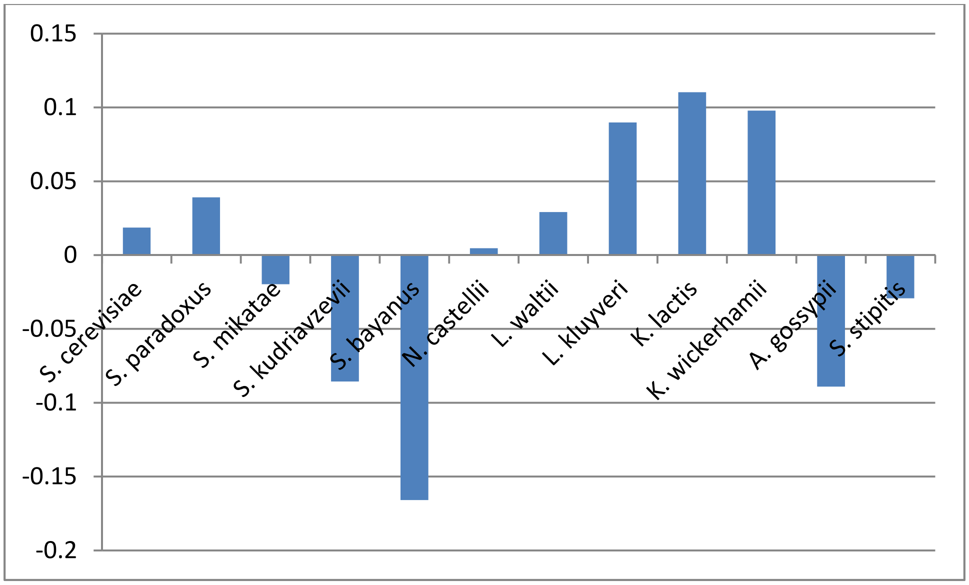

Figure S6 Differential Hi-C efficiency rates by species for the M-Y sample. For each species, the Hi-C efficiency rate was calculated as $E_{species} = \frac{f_{species}(\text{Hi-C})}{f_{species}(\text{shotgun})}$, where $f_{species}$(library) is the fraction of reads from a sequencing library that align to the given species' reference genome. These efficiency rates were log-scaled and then normalized to create an average of 0 over all species.
