# Supplementary material for: Species-Level Deconvolution of Metagenome Assemblies with Hi-C–Based Contact Probability Maps
Source: G3 (Bethesda). 2014 May 22;4(7):1339–46. doi: 10.1534/g3.114.011825 (PMC4455782; doi:10.1534/g3.114.011825)
Supplement: Supporting Information [file supp_g3.114.011825_FigureS7.pdf]

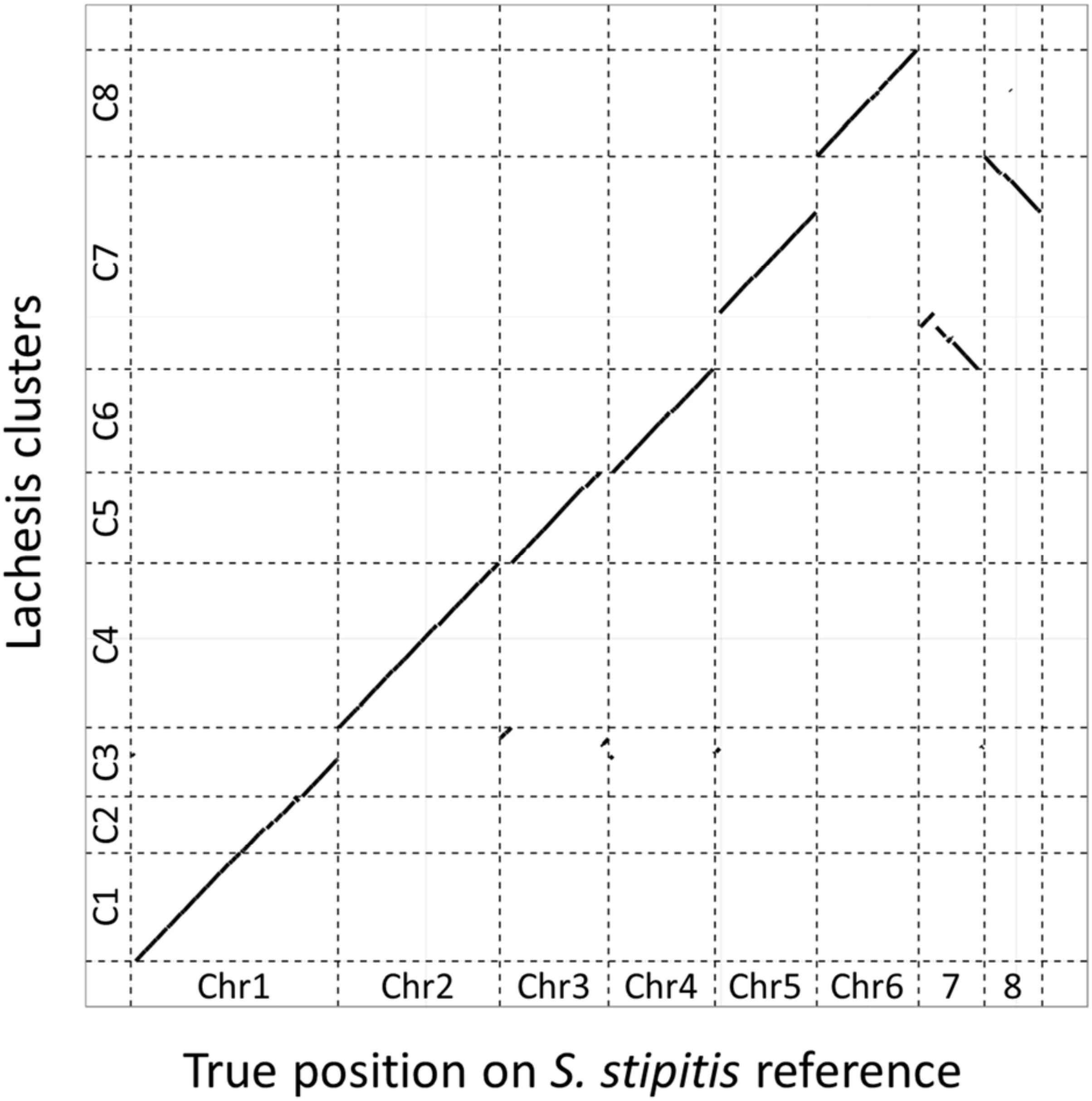

Figure S7 Accuracy of Lachesis assembly of *Scheffersomyces stipitis*. The contigs in the MetaPhase cluster corresponding to *S. stipitis* were clustered, ordered, and oriented with Lachesis (Burton *et al.* 2013) (Figure 2C). Shown here is a validation of the Lachesis assembly. Every contig that is placed by Lachesis and which aligns to the *S. stipitis* reference genome is shown. *x*-axis: the contig's true position in the *S. stipitis* reference. *y*-axis: the contig's placement in the Lachesis assembly (note that both the order of the clusters on the *y*-axis and the overall orientation of each cluster are arbitrary; they are chosen here for visual clarity and are not the same as in Figure 2C.)
