# Supplementary material for: Species-Level Deconvolution of Metagenome Assemblies with Hi-C–Based Contact Probability Maps
Source: G3 (Bethesda). 2014 May 22;4(7):1339–46. doi: 10.1534/g3.114.011825 (PMC4455782; doi:10.1534/g3.114.011825)
Supplement: Supporting Information [file supp_g3.114.011825_FigureS8.pdf]

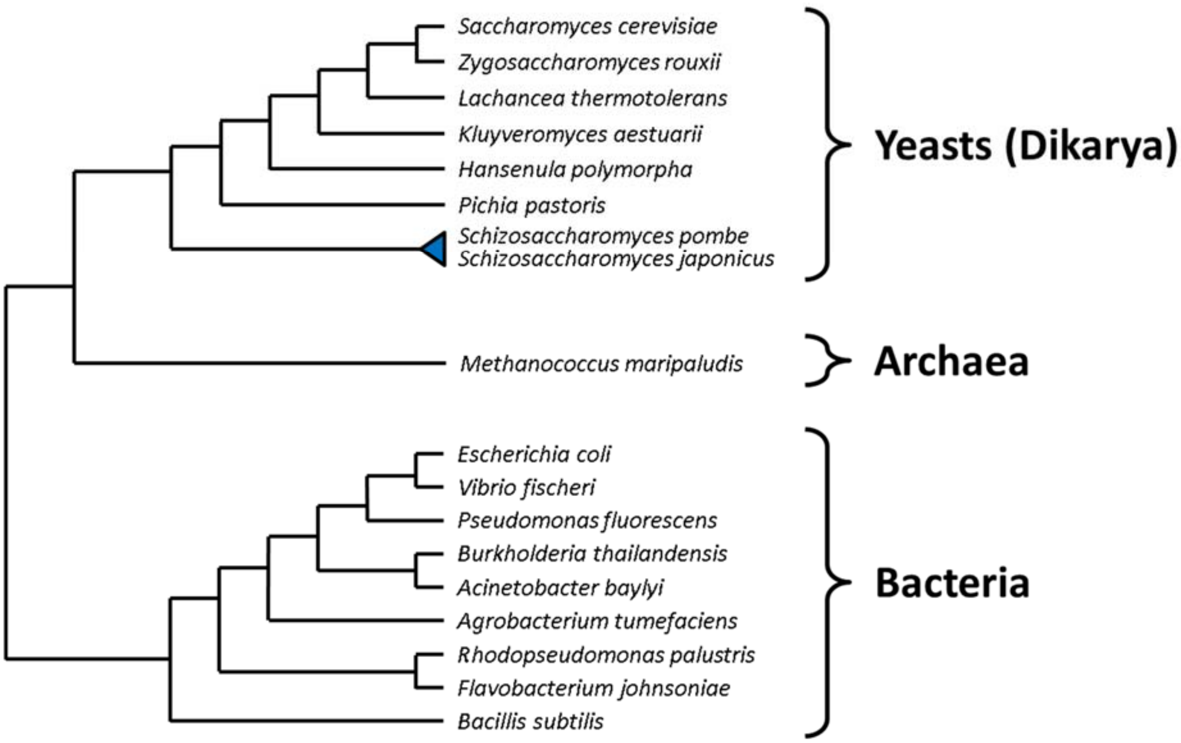

Figure S8 M-3D species phylogeny. Phylogenetic tree of the 18 yeast, archaeal, and bacterial strains used in the M-3D sample (Table S2).
