# Supplementary material for: Species-Level Deconvolution of Metagenome Assemblies with Hi-C–Based Contact Probability Maps
Source: G3 (Bethesda). 2014 May 22;4(7):1339–46. doi: 10.1534/g3.114.011825 (PMC4455782; doi:10.1534/g3.114.011825)
Supplement: Supporting Information [file supp_g3.114.011825_FigureS9.pdf]

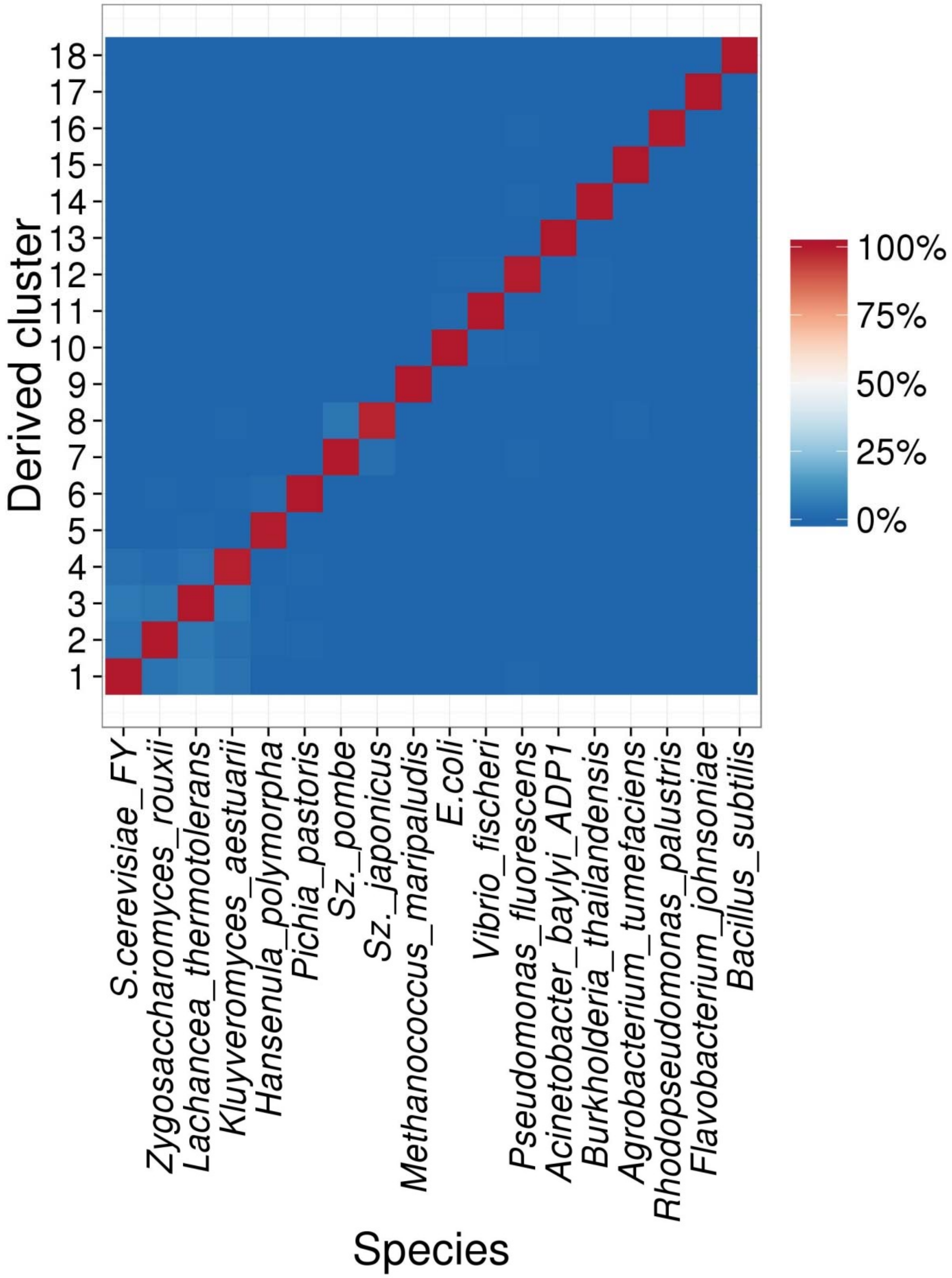

Figure S9 Heatmap of non-unique reference alignments of contigs in each M-3D cluster. This is identical to Figure 3B, except that all contig alignments to all genomes are shown here, whereas in Figure 3B only contigs that align uniquely to a single reference genome are shown.
