# Supplementary material for: Species-Level Deconvolution of Metagenome Assemblies with Hi-C–Based Contact Probability Maps
Source: G3 (Bethesda). 2014 May 22;4(7):1339–46. doi: 10.1534/g3.114.011825 (PMC4455782; doi:10.1534/g3.114.011825)
Supplement: Supporting Information [file supp_g3.114.011825_FigureS10.pdf]

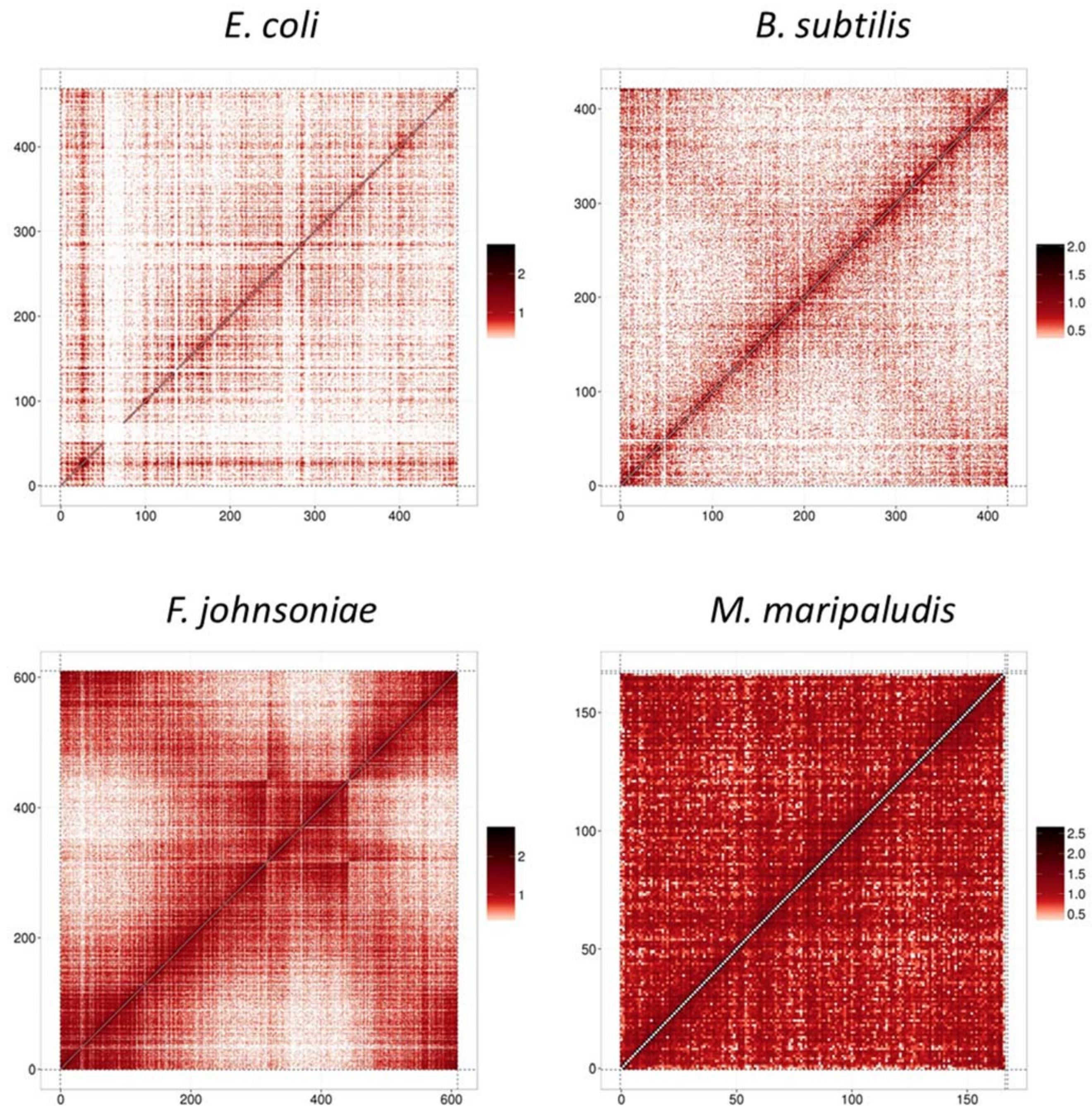


**Figure S10** Heatmaps of M-3D Hi-C links aligned to prokaryotic reference genomes. Reads from the M-3D *Hin*dIII non-resuspended library (Table S3) were aligned to the draft assemblies of four prokaryotic species present in the M-3D sample. Each heatmap has a resolution of 10 Kb, and the legend indicates the $\log_{10}$ of link density.
